# Formation of functional CENP-B boxes at diverse locations in repeat units of centromeric DNA in New World monkeys

**DOI:** 10.1038/srep27833

**Published:** 2016-06-13

**Authors:** Kazuto Kugou, Hirohisa Hirai, Hiroshi Masumoto, Akihiko Koga

**Affiliations:** 1 Department of Frontier Research, Kazusa DNA Research Institute, Kisarazu 292-0818, Japan; 2 Primate Research Institute, Kyoto University, Inuyama 484-8506, Japan

## Abstract

Centromere protein B, which is involved in centromere formation, binds to centromeric repetitive DNA by recognizing a nucleotide motif called the CENP-B box. Humans have large numbers of CENP-B boxes in the centromeric repetitive DNA of their autosomes and X chromosome. The current understanding is that these CENP-B boxes are located at identical positions in the repeat units of centromeric DNA. Great apes also have CENP-B boxes in locations that are identical to humans. The purpose of the present study was to examine the location of CENP-B box in New World monkeys. We recently identified CENP-B box in one species of New World monkeys (marmosets). In this study, we found functional CENP-B boxes in CENP-A-assembled repeat units of centromeric DNA in 2 additional New World monkeys (squirrel monkeys and tamarins) by immunostaining and ChIP-qPCR analyses. The locations of the 3 CENP-B boxes in the repeat units differed from one another. The repeat unit size of centromeric DNA of New World monkeys (340–350 bp) is approximately twice that of humans and great apes (171 bp). This might be, associated with higher-order repeat structures of centromeric DNA, a factor for the observed variation in the CENP-B box location in New World monkeys.

The centromere is the part of a chromosome to which spindle fibers attach via the kinetochore and serves as the forefront of chromosome migration during cell division. CENP-B is involved in centromere formation^1^, and its amino acid sequence is highly conserved in a wide range of mammals^2^,^3^. The proposed function of CENP-B is that its binding to DNA induces and stabilizes processes of centromere formation^4^,^5^,^6^,^7^,^8^. CENP-B contains a DNA binding domain at its N terminus and recognizes a nucleotide motif called the CENP-B box^9^. CENP-B box was originally identified as a 17-bp-long motif (YTTCGTTGGAARCGGGA) and subsequently shown to function as long as the core recognition sequence consisting of the 9 underlined nucleotides is present^10^. We use the term CENP-B box in its broad sense: a 17-bp nucleotide motif containing the core recognition sequence (NTTCGNNNNANNCGGGN). The centromere region of chromosomes usually contains large amounts of tandem repeat DNA, and CENP-B box occupies part of their repeat units. Humans and mice carry CENP-B boxes in their centromeric repetitive DNA of all autosomes and the X chromosome^9^.

Since its original discovery in humans and mice^9^, CENP-B box has been identified in great apes (chimpanzees, bonobos, gorillas, and orangutans)^11^, and wallabies^12^. Computational analyses of genome sequence databases have found sequence blocks that are similar to CENP-B box in some mammalian species, including horses, dogs and elephants^13^. However, experimental analyses that determine if these blocks function as CENP-B box have yet to be performed. The location of CENP-B box in the repeat units of centromeric DNA in the great apes is identical to that in humans^11^. This uniform location might be a result of functional constraints or evolutionary processes. With respect to functional constraints, even if a CENP-B box sequence is formed by mutations in centromeric DNA, it may not function or may function poorly when it arises at a different position in the repeat units. The evolutionary process explanation suggests that the CENP-B boxes present in extant hominid species (humans and great apes) may have been derived from a CENP-B box that occurred in their common ancestor and/or from CENP-B boxes that emerged recurrently at this particular position in multiple lineages. These explanations are not testable by analyzing CENP-B boxes of hominids.

Because CENP-B box is a nucleotide block consisting of as few as 9 core nucleotides, it can be assumed that CENP-B box sequences are frequently formed by chance at various positions within repeat units by mutations in centromeric DNA. Observations that functional CENP-B boxes are located at different positions in a taxon would be indirect evidence against the first explanation (functional constraints) and would support the second explanation (a common origin and/or recurrent emergences at a single site) at least in hominids. The purpose of the present study was to find such an example, if any, in New World monkeys. Before examining this in New World monkeys, we tried to find an example in mice because CENP-B box has been found in the Asian mouse (*Mus caroli*)^14^ in addition to the house mouse (*M. musculus*)^9^. However, comparisons of the positions could not be conducted because of the difficulty in establishing accurate nucleotide alignments of the repeat units of the centromeric DNA of these two species. We recently identified a functional CENP-B box in the common marmoset, which is a New World monkey^15^. New World monkeys share alpha satellite DNA as their major centromeric DNA, and the sequences of the repeat units can be aligned among the species so far examined^16^,^17^,^18^,^19^,^20^. In this study, we designed and conducted experiments to find CENP-B boxes occurring at multiple locations.

First, by immunofluorescence staining of cultured cells, we observed the co-localization of CENP-A and CENP-B accumulation signals in 4 of the 6 New World monkey species examined. CENP-A is a centromere-specific conserved histone H3 variant, and accumulation signals indicate the locations of functional centromeres^21^. Next, we examined the nucleotide sequences of alpha satellite repeat units, and found CENP-B box sequences at moderate frequencies in repeat units of 3 of the 4 species. Alignment of their repeat unit sequences revealed that the locations of the CENP-B boxes differed from one another. We then performed a ChIP-qPCR analysis and demonstrated the presence of the CENP-B box in alpha satellite repeat units that were enriched with CENP-A antibodies. Thus, these results provide molecular evidence for the existence of multiple, functional CENP-B boxes.

## Results

### CENP-B assembly at centromere revealed by immunofluorescent staining

**Figure 1:**
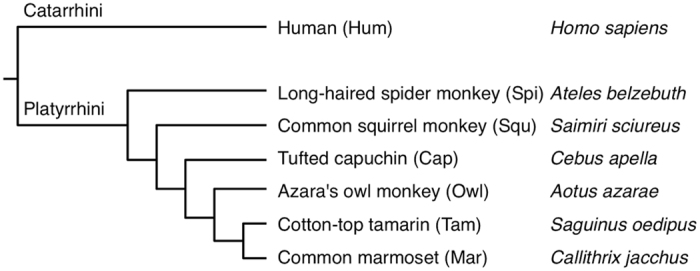
Phylogenetic relationships between 6 New World monkey species used in this study. For each species, the common name, three-letter abbreviation used in this report, and scientific name are shown. Phylogenetic relationships were determined to reflect phylogenetic trees widely accepted in the latest primate taxonomy^26^,^27^.

To find species that might harbour CENP-B boxes in alpha satellite DNA, we conducted immunofluorescent cell staining assays for 6 New World monkeys: capuchin (Cap), marmoset (Mar), owl monkey (Owl), spider monkey (Spi), squirrel monkey (Squ), and tamarin (Tam). Names in full are listed in Fig. 1, along with the proposed phylogenetic relationships. Human HeLa cells were treated as positive controls. An antibody against CENP-B was used in the assays. An antibody against CENP-A was also used as a marker for functional centromere locations. CENP-A is a centromere-specific conserved histone H3 variant^21^.

**Figure 2:**
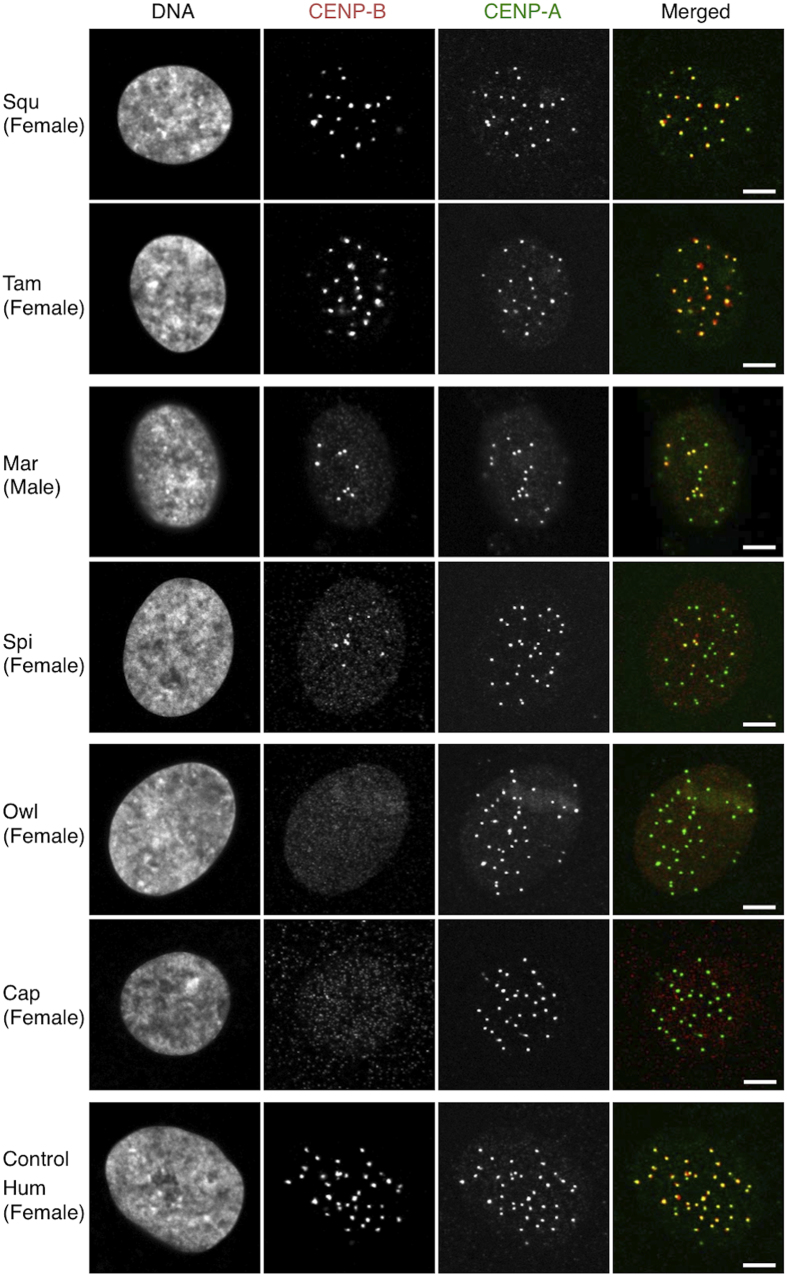
Results of immunofluorescent cell staining analysis. Cells were co-stained with antibodies against CENP-A (green) and CENP-B (red). DNA was stained with DAPI. Scale bar, 5 μm.

Photographs of typical cells are shown in Fig. 2. In all seven species, distinct speckled signals for CENP-A were observed. Human cells exhibited clear speckled signals for CENP-B, which all overlapped with CENP-A signals, consistent with past results repeatedly obtained, including those in our previous study^15^. The same situation was observed in Squ (99.7% overlap, n = 11) and Tam (100% overlap, n = 5) cells. Speckled CENP-B signals also appeared in Mar and Spi cells, but the number of these signals was smaller than that for CENP-A. In Mar cells, CENP-B signals overlapped with approximately 30% CENP-A signals, similar to our previous results^15^, and therefore demonstrating the high reproducibility of our methods. In Spi cells, speckled CENP-B signals appeared to overlap with CENP-A signals, but the number of overlapping signals was slightly lower than that in Mar cells. No clear speckled CENP-B signal overlapping with the centromere was detected in Cap and Owl cells.

### Detection of CENP-B box sequences in alpha satellite DNA

We next performed sequencing analyses of alpha satellite DNA of the 6 New World monkeys for detection of CENP-B box sequences. Well-established genomic sequence databases are available for some of the 6 species, but it is widely thought that repetitive DNA regions contain many sequencing errors and assembly errors, especially in databases based on next-generation sequencing. Trace archive collections of sequence data produced using the Sanger method are thought to be highly reliable. However, results of data collection from trace archives are highly dependent on the query sequence used, making it difficult to estimate the frequency of CENP-B box accurately. For these reasons, we collected alpha satellite DNA clones from genomic libraries using our own methods to avoid bias. From genomic libraries of the 6 species (vector, fosmid pCC1FOS; insert 40- to 44-kb genomic DNA fragments produced by mechanical shearing), we collected fosmids containing high-copy-number repetitive DNA by our modified genomic hybridization method^22^. We sequenced one end region of these clones by the Sanger method, using a universal primer, which provided sequence reads of >800 bp. We then compared the obtained sequence reads with the consensus sequence of Mar alpha satellite DNA^22^ and considered those showing >60% nucleotide identity over a 400-bp region to be alpha satellite DNA. From each sequence read, partial repeat units located at terminal regions were excluded and full-length repeat units present in the internal region were cut out and collected. One or two full-length repeat units were obtained from each clone, and this data collection continued until the total number of repeat units exceeded 40. All sequence reads used for our analyses were deposited in GenBank with the following accession numbers: Cap, LC075856-LC075884; Mar, LC075851-LC075855; Owl, LC075885-LC075899, AB761997-AB762011; Spi, LC075900-LC075927; Squ, LC075928-LC075953; Tam, LC075954-LC075981.

**Figure 3:**
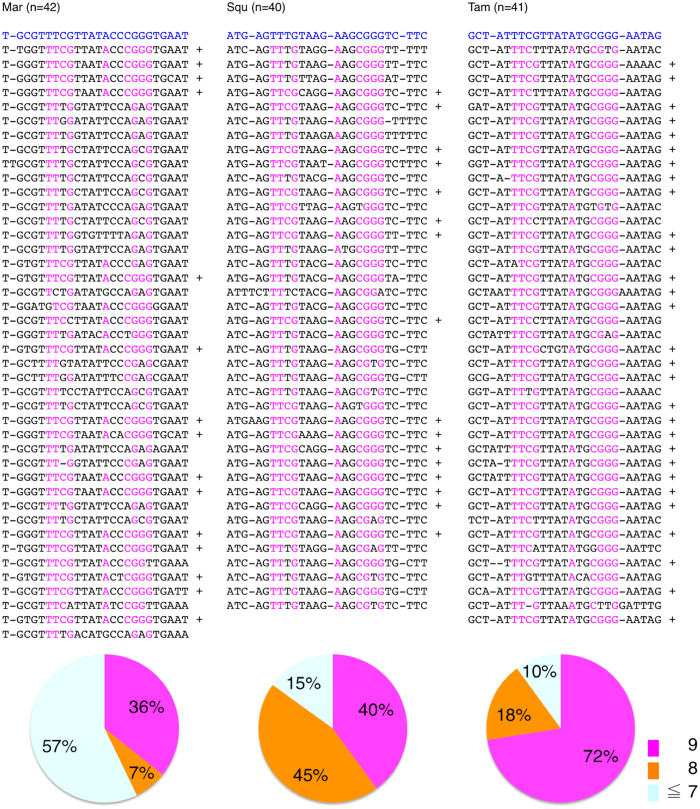
Distribution of CENP-B box sequences on alpha satellite repeat units. Alignments of entire alpha satellite repeat units of the 6 species are shown in supplementary Figure S1. CENP-B boxes were found in 3 (Mar, Squ and Tam) of the 6 species. For these 3 species, sequences of CENP-B boxes and their flanking regions are cut out and shown in this figure. For Squ and Tam, sequences of the strand opposite to the strand displayed in supplementary Figure S1 are shown here because CENP-B box sequences were located on these opposite strands. Nucleotides that match those in the CENP-B box are colored magenta. The asterisk indicates complete matching (at all 9 nucleotide sites) with the CENP-B box sequence. Pie charts illustrate frequencies of repeat units containing 9, 8 and lower numbers of matching nucleotides.

Alignment of the sequences of entire repeat units is shown in supplementary Figure S1. CENP-B box sequences were identified in more than one repeat unit of Mar (15/42; 36%), Squ (15/40; 40%) and Tam (29/41; 72%), and were not found in any repeat units of Cap, Owl and Spi (n = 41, 40, 41, respectively). Thus, of the 4 species that showed CENP-B signals overlapping centromeres in our immunofluorescent cell staining assays, 3 were found to carry CENP-B box sequences at moderate frequencies. For these 3 species, regions corresponding to the CENP-B box sequences and their flanking sequences are shown in Fig. 3.

The frequency of CENP-B box sequences in alpha satellite repeat units of Mar, Squ and Tam was not strongly correlated with the number of CENP-B immunofluorescence signals (relative to that of CENP-A signals). In addition, a CENP-B box sequence was not found in Spi. However, a fact to be taken into consideration is that CENP-B boxes are, in humans, not uniformly distributed but concentrated in specific regions of alpha satellite DNA^9^,^23^,^24^. As our frequency estimates were obtained from limited numbers of alpha satellite repeat units, they may have been affected by uneven distributions of CENP-B boxes in alpha satellite DNA of New World monkeys.

**Figure 4:**
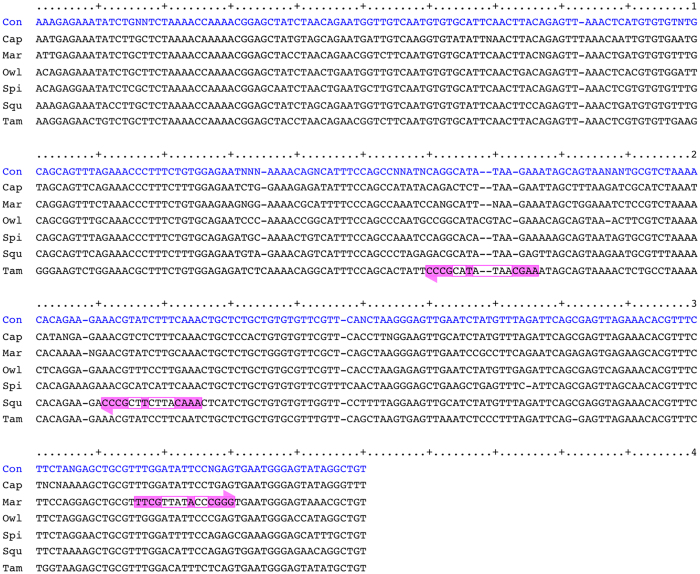
Locations and directions of CENP-B boxes found in New World monkeys. The within-species consensus sequences were aligned among the 6 species, and 9 nucleotide sites corresponding to those in the CENP-B box are indicated by magenta boxes. The right-pointing and left pointing arrows indicate that CENP-B boxes are located on the upper strand and lower strand, respectively.

The within-species consensus sequences of alpha satellite repeat units were well aligned among the 6 species, as shown in Fig. 4. We denoted the CENP-B boxes found in Mar, Squ and Tam as boxMar, boxSqu and boxTam, respectively. The alignment showed that the locations of these 3 boxes differed from one another. Moreover, boxSqu and boxTam were located on the strand opposite to the strand carrying boxMar. In addition, it is notable that the region occupied by boxTam includes an extra nucleotide site (the 4th site from the right end) as compared with the corresponding region in the consensus sequence among the 6 New World monkeys.

### CENP-B box specific CENP-B assembly determined by ChIP-qPCR

**Figure 5:**
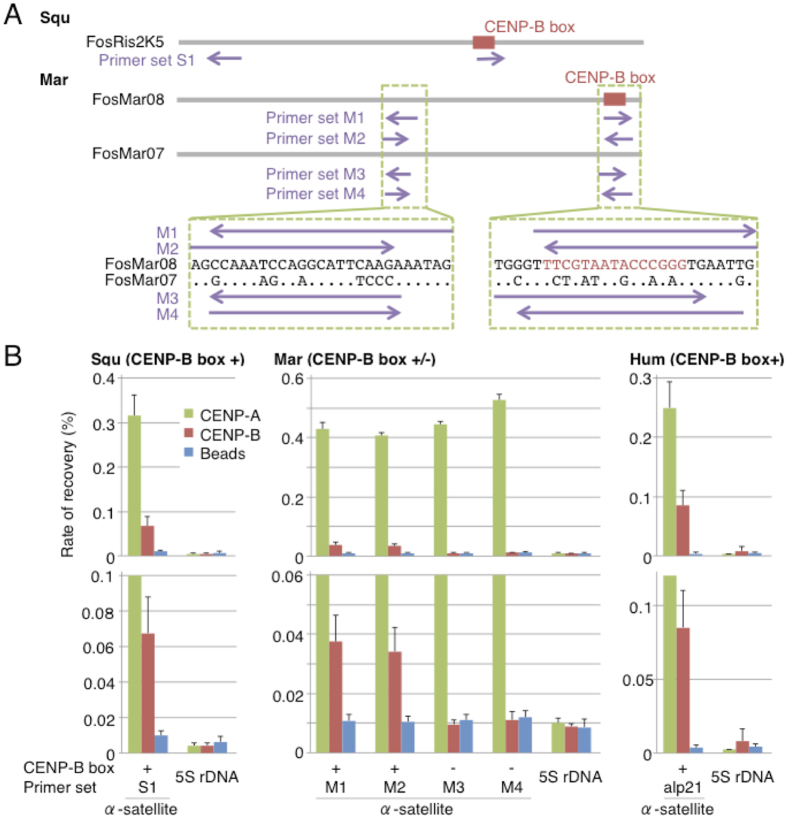
Binding of CENP-A and CENP-B to alpha-satellite DNA. (**A**) Primer positions used in ChIP-qPCR analyses. A grey horizontal bar and a red box represent one repeat unit of alpha satellite (about 340 bp) and CENP-B box, respectively. Positions of primers used in (**B**) are indicated with purple arrows. Green boxed regions were magnified and shown with consensus nucleotide sequences of each clone. A dot indicates that the nucleotide sequence is the same as in the upper sequence. FosMar08 (GenBank LC030305) and FosMar07 (LC076441 and LC076442) are fosmid clones sequenced and characterized in our previous study^20^. FosMar08 contained the CENP-B box in 17 of the 38 repeat units, and FosMar07 did not contain the CENP-B box in any of its 6 repeat units. We prepared qPCR primers to represent the consensus sequence of the 17 CENP-B box-containing repeat units of FosMar08, or all repeat units of FosMar07. (**B**) Relative numbers of DNA recovered with anti-CENP-A or anti-CENP-B antibody or without antibody (beads for control) were analyzed by real-time PCR with the primer sets in (**A**). The 5S rDNA locus was a negative control. Rate of recovery was calculated as a percentage of recovered DNA compared to input DNA. Error bars, s.d. (n = 3).

To demonstrate that CENP-B binds to centromeric alpha satellite DNA containing CENP-B box sequences in New World monkeys, we performed ChIP-qPCR analyses of CENP-A and CENP-B by using the primer sets indicated in Fig. 5A. As described earlier, CENP-B signals overlapped with all centromeric regions in Squ and Tam cells, and with some centromeric regions in Mar and Spi cells (Fig. 2). We selected Squ and Mar cells from each type of CENP-B signals (all or some overlapping, respectively) for ChIP-qPCR analyses.

In Squ cells, both CENP-A and CENP-B enriched alpha satellite repeats, but did not enrich the control 5S rDNA site (Fig. 5, primer set S1), similar to the case of the chromosome 21 higher-order repeat (HOR) alpha satellite DNA, which contained CENP-B box and the control site in human cells (Fig. 5, primer set alp21). Thus, both CENP-A and CENP-B assemble in the same alpha satellite repeats in Squ centromeres. In Mar cells, we could obtain relatively long alpha satellite repeat sequences in FosMar08 (38 units) and FosMar07 (6 units) clones previously sequenced^20^. CENP-B boxes were found only in the former clone. CENP-A enriched both Mar alpha satellite repeats regardless of whether it contained a CENP-B box or not, as observed by the ChIP-qPCR analysis (Fig. 5, primer sets M1-M4). This result indicates that centromeric CENP-A chromatin assembles both on FosMar08 and FosMar07 alpha satellite repeats. In contrast, CENP-B enriched only FosMar08 alpha satellite repeats containing CENP-B boxes (Fig. 5, primer sets M1 and M2). Lower enrichment levels of CENP-B, especially in Mar cells as compared to human cells, may be due to a limited number of interacting molecules (CENP-B proteins and/or boxes). Thus, our ChIP-qPCR analysis provided clear evidence that CENP-B binds only to centromeric alpha satellite repeats containing CENP-B boxes both in Mar and Squ cells, whereas the centromeric CENP-A chromatin assembles on alpha satellite repeats regardless of the existence or absence of CENP-B boxes in Mar cells.

## Discussion

In humans, CENP-B is involved in centromere functions, such as *de novo* CENP-A chromatin assembly^4^,^5^,^6^, CENP-A nucleosome stabilization^7^, and fidelity enhancement of chromosome segregation^8^. CENP-B functions by binding to centromeric DNA at a CENP-B box^9^. In the present study, we identified CENP-B box sequences and demonstrated their function as CENP-B binding motifs in 3 New World monkey species, including one that was previously reported. We obtained molecular evidence for a functional link between the CENP-B box sequences and the centromere-determined chromatin via the CENP-B/CENP-B box interaction. First, immunofluorescence cell staining assays showed overlap between speckled CENP-B signals and CENP-A signals, the latter of which indicate the formation of centromere-specific nucleosomes. Second, the ChIP-qPCR analysis revealed CENP-B binding to alpha satellite repeat units that were associated with CENP-A chromatin in a CENP-B box-dependent manner.

A sequence comparison of the repeat units revealed that boxMar, boxSqu, and boxTam were located at different positions. In addition to positional differences, the CENP-B boxes were located on different strands and varied in length. The observation of multiple CENP-B boxes in the New World monkeys raised the question of why an additional CENP-B box is not found in hominids. One possibility is that the number of CENP-B boxes reached, at a certain time in the past, the maximum amount the genome can contain, by random genetic drift and/or natural selection. This explanation requires the assumption that 2 or more CENP-B boxes cannot exist in a single repeat unit. This is likely to be true, considering that the array of CENP-B boxes along alpha satellite DNA is highly associated with higher-order repeat structures in humans^10^,^23^,^24^. Higher-order repeat structures are organization of repeat units in which a block of multiple repeat units forms a larger repeat unit and larger repeat units are repeated in tandem. A new CENP-B box sequence that emerges in an established CENP-B box array may disturb this array and have a tendency to be excluded.

The repeat unit size of alpha satellite DNA of New World monkeys (340–350 bp) is approximately twice that of hominids (171 bp). This may have significance for the observed location variation in New World monkeys. As described earlier, the array of CENP-B boxes is highly associated with higher-order repeat structures in humans. Although several array patterns have been observed, those with an “every other monomer scheme” are likely to be common^5^,^23^. The “scheme” refers to the alternate appearance of a repeat unit that carries a CENP-B box and a unit that is free of a CENP-B box. Because the repeat unit size of alpha satellite DNA of New World monkeys is approximately twice the size of humans, a simple repetition of CENP-B-box-carrying units in New World monkeys is nearly equivalent, with respect to the intervals between CENP-B boxes, to the CENP-B box arrays common in humans (the alternating units). The association with higher-order repeat structures may be essential for the functioning of CENP-B boxes in hominids, but this association may not be strictly required in New World monkeys. These less stringent requirements may lead to a higher chance of expansion of a newly formed CENP-B box in New World monkeys than in hominids.

Two evolutionary scenarios may explain the variation in the location of CENP-B boxes among New World monkeys. In one scenario, the CENP-B boxes emerged via different mutations that occurred after lineage divergence. In the alternative scenario, the genomes of the common ancestor contained boxMar, boxSqu, and boxTam, and these were inherited by Mar, Squ, and Tam, respectively, as their major CENP-B boxes. These two scenarios are not mutually exclusive but can be considered simple cases. At present, we do not have sufficient information to support either scenario. It is important to determine whether boxMar, boxSqu, and boxTam are unique to the Mar, Squ, and Tam genomes, respectively; however, this cannot be determined at present owing to the limited sample sizes. Although it is likely that boxMar, boxSqu, and boxTam are major CENP-B boxes in their respective host species, boxMar may exist in Squ or Tam, boxSqu in Mar or Tam, and boxTam in Mar or Squ. This would be resolved by a thorough survey of repetitive sequences, which may become available through the development of sequencing techniques and analysis algorithms.

## Methods

### Ethics statement

All animal experiments in this study were approved by the Animal Care and Use Committee of Kyoto University Primate Research Institute (KUPRI), and were performed in accordance with the Guidelines for Care and Use of Nonhuman Primates (Version 3; June 2010), published by KUPRI.

### Animals

With all 6 New World monkey species, cultured epithelial cells were used for immunofluorescent staining analysis, as well as as sources of genomic DNA for genomic library construction and subsequent cloning and sequencing analyses. Tissue samples for cell culture were collected from the body skin of a dead animal or a tiny piece of skin from the ear of a live animal anesthetized for other purposes, such as a medical treatment or health checkup.

The animals from which tissue samples were collected were all bred at KUPRI. Their individual identification number and sex were as follows: Cap, Ca10, female; Mar, 186, male; Owl, A34, female; Spi, ABE1, female; Squ, 116, female; Tam, 219, female.

### Cell culture

Cells of New World monkeys were cultured in AmnioMAX-II Complete Medium (Life Technologies). Human HeLa cells were cultured in D-MEM High Glucose (Wako) supplemented with 10% FBS and penicillin-streptomycin. These cells were incubated in a humidified incubator set at 37 °C and 5% CO_2_.

### Antibodies

Rat monoclonal anti-human CENP-A antibody (6F2, gifted by Kinya Yoda)^25^ and mouse monoclonal anti-human CENP-B antibody (5E6C1)^25^ were used in this study. Alexa Fluor 594 conjugated goat anti-rat IgG and Alexa Fluor 488 conjugated goat anti-mouse IgG were used as secondary antibodies in cell staining. Specificity of the anti-human CENP-A antibody to New World monkey CENP-A was confirmed by western blotting (supplementary Figure S2). The anti-human CENP-B antibody was considered to recognize CENP-B of New World monkeys used in this study because amino acid sequences of all reported New World monkey CENP-B exhibit high conservation to human CENP-B: 98.3% identity in Owl, 98.2% in dusky titi, 98.0% in Squ, 97.8% in Spi, and 96.6% in Mar^3^.

### Cell staining

Immunofluorescence staining of CENP-A and CENP-B was performed as described previously^25^. Staining images were acquired using an LSM700 microscope (Zeiss) equipped with Alpha Plan-Apochromat 63 × /Oil M27 lens (Zeiss). Three slices of Z-stacks at 0.34 μm intervals were displayed as maximum intensity projection. To analyze co-localization between CENP-A and CENP-B, Z-stacks at 0.22 μm intervals were acquired to cover all signals of CENP-A and CENP-B.

### Chromatin immunoprecipitation

Cells were trypsinized and harvested in a centrifuge tube. Cells were washed in PBS and fixed with 0.5% formaldehyde at room temperature for 10 minutes. After the reaction was stopped by addition of glycine to a final concentration of 125 mM, the cells were used for ChIP as described previously^6^,^7^ with some modifications. In brief, fixed cells were suspended in sonication buffer (20 mM Tris-HCl pH 8.0, 1 mM EDTA, 1 mM DTT, 0.02% SDS, cOmplete ULTRA EDTA free [Roche]), and sonicated with Bioruptor (Cosmobio) to fragment chromatin DNA to an average size of 200–500 bp. The soluble chromatin was recovered by centrifugation, diluted with twice volume of IP buffer (30 mM Tris-HCl pH8.0, 450 mM NaCl, 0.75 mM EDTA, 0.75 mM DTT, 1.5% Triton X-100, 0.075% SDS, 7.5% glycerol, cOmplete ULTRA EDTA free), and immunoprecipitated using anti-CENP-A antibody or anti-CENP-B antibody pre-incubated with Dynabeads-Protein G (Life Technologies). ChIP DNA and input DNA were purified with MinElute PCR Purification kit (QIAGEN) after Proteinase K treatment and incubation at 65 °C to de-crosslink. The purified DNA was analyzed by real-time PCR (Bio-Rad) with SYBR Premix Ex Taq II (Takara Bio) and primer pairs indicated below.

Primer set S1

5′- CGCTTCTTACGAACTCATCTGC-3′ and

5′- GCTAGATAGCTCCGTTTTGGTTTTAG-3′

Primer set M1

5′- CTATTTCTTGAATGCCTGGATTTGG-3′ and

5′- TTTCGTAATACCCGGGTGAATTG-3′

Primer set M2

5′- AGCCAAATCCAGGCATTCAAG-3′ and

5′- CAATTCACCCGGGTATTACGAA-3′

Primer set M3

5′- TGCGTTCTGATATGCCAGAGTG-3′ and

5′- TGGGAAATGCTTGCTTTTGC-3′

Primer set M4

5′- GCAAAAGCAAGCATTTCCCA-3′ and

5′- CATTCACTCTGGCATATCAGAACG-3′

Primer set 5S rDNA

5′- GTCTACGGCCATACCACCC-3′ and

5′- GCCTACAGCACCCGGTATTC-3′

Primer set alp21 25

5′-CTAGACAGAAGCCCTCTCAG-3′ and

5′-GGGAAGACATTCCCTTTTTCACC-3′

Primer set 5S rDNA (Hum)

5′-CCGGACCCCAAAGGCGCACGCTGG-3′ and

5′-TGGCTGGCGTCTGTGGCACCCGCT-3′

### Cloning and sequencing experiments

Genomic libraries were constructed, using genomic DNA extracted from cultured cells. These libraries were screened for clones containing alpha satellite DNA as inserts. The clones obtained were sequenced by the Sanger method. All these experiments were conducted as described previously^18^,^19^,^22^.

## Additional Information

**How to cite this article**: Kugou, K. *et al*. Formation of functional CENP-B boxes at diverse locations in repeat units of centromeric DNA in New World monkeys. *Sci. Rep.* **6**, 27833; doi: 10.1038/srep27833 (2016).

## Supplementary Material

Supplementary Information
