# Supplementary material for: Formation of functional CENP-B boxes at diverse locations in repeat units of centromeric DNA in New World monkeys
Source: Sci Rep. 2016 Jun 13;6:27833. doi: 10.1038/srep27833 (PMC4904201; doi:10.1038/srep27833)
Supplement: Supplementary Information [file srep27833-s1.pdf]

Supplementary information

for

Title: Formation of functional CENP-B boxes at diverse locations in repeat units of centromeric DNA in New World monkeys

Authors: Kazuto Kugou, Hirohisa Hira, Hiroshi Masumoto & Akihiko Koga

Supplementary figures

Figure S1. Alignments of entire alpha satellite repeat units of the 6 species. The alignment was performed with the MEGA6 program (Tamura *et al*. 2013) under default settings. Names of fosmid clones are listed on the left of the alignment. See additional information in the legend to Figure 1. "Con" indicates the consensus sequence.

Tamura, K., Stecher, G., Peterson, D., Filipski, A. & Kumar, S. MEGA6: Molecular Evolutionary Genetics Analysis version 6.0. *Mol. Biol. Evol.* **30**, 2725-2729 (2013).

Figure S2. Western blotting analysis of CENP-A in New World monkeys. Whole-cell extracts were prepared and analyzed by western blotting using the anti-human CENP-A antibody (6F2).

Cap (n=41). 1 of 2

```
                 .........+.........+.........+.........+.........+.........+.........+.........+.........+.........1
            Con  AATGAGAAA-TATCTTGCTCTAAAAC--AAAAACGG-AGCTATGT-AGCAGAATGA-TTGTCAAGG-TGTATATT?AA-CTTACAGAG-TTTAAACAATT
Cap1G6a     01   AATGAGAAA-TATCTGGCTCTATAAC-AAAA-GCGG-AACTATGG-AGCAGAATGA-CTATCAAGG-TGTATATTCAA-CATAGAGAG--TTAAACAGAT
Cap1I4a     02   AATGAGAAA-TGTATGTCTCTAAAAC--CAAAACGG-AGCTATCT-AACAGAATGA-TTGTCAAGG-TGTATATTAAACTTAACACAG--TTAAACAATT
Cap1I4b     03   AATGAGAAA-TATCTGGTTCTATAAC-AAAA-GCGG-AACTATGT-AGCAGAATGA-CTATCAAGG-TGTATATTCAA-CATAGAGAG--TTAAACAGAT
Cap1I8a     04   AATGAGAAA-TATCTGGCTCCAAAAC--AATAACGG-AACTATGT-AGCAGAATGA-TTGTCTAGG-TGTATATTAAA-CTTACAGAG--TTAAACAATT
Cap1J2a     05   AATCAGAAA-CATCTTGCTCCAAAAAG-AAAAACGC-AGCTTTGT-AGGAGAGTGG-TTGTCAAGC-TGTACAATCAA-CTTACAGAG-TT-AAAGTGAG
Cap1J2b     06   AATCAGAAA-CATCTTGCTCTAAAAC--ATAAGCAC-AGCTATCT-AGCAGAATGG-TTGTCAAGC-TGTGAAATCAT-CTTATGGAG-TG-AAATTGAT
Cap1J4a     07   AATGAGAAA-TATCTGGTTCAAATAAACAAAAACGG-AGCTCTGT-AGCAGAATGC-TTGTCAACG-TGTTTATTAAA-CTTACAGAG--TTAAACAATT
Cap2A4a     08   AATGAGAAA-TATCTGGCTCTAAAAC--AAAAACGG-AGGTATGT-AACAGAATGA-TTGTCAAGG-TGTATATTAAT-CTTACAGAG-TTAAA-CAATT
Cap2A6a     09   AATGAGAAA-TATCTGGCTCTATAAC-AAAA-GCGG-AACTATGG-AGCAGAATGA-CTATCAAGG-TGTATATTCAA-CATAGAGAG--TTAAACAGAT
Cap2C4a     10   ATTGAGAAA-TATCTGGTTCTATAAC-AAAAAGCGG-AACTATGT-AGCAGAATGA-CTATCAAGG-TGTATATTCAA-CATAGAGAG--TTAAACAGAT
Cap2D2a     11   AATCAGAAA-CATCTTGCTCCAAAAAG-AAAAACGC-AGCTTTGT-AGGAGAGTGG-TTGTCAAGC-TGTACAATCAA-CTTACAGAG-TT-AAAGTGAG
Cap2D4a     12   ATTGAGAAA-TTTCTTGCTTTAAAACCAAAAACCGG-ACCTATGTTAACAGAATGT-TTGTCAAGGGTGTATATTTAAACTTACCGGGGTTAAAACAATT
Cap2D4b     13   AAAGAGAAA-TATCTGGGTCTAAAAC--AAAAACGG-AGCTATGT-AACAGAATGA-TTGTCATGG-TGAATATT-AAAGTTACAGAG--TTAAACAATT
Cap2F8a     14   AATGAGAAA-TATCTTGATCTAAAAC--AAAAACGC-AGCTCTCT-AGCTGAATGG-TAGTCAAGC-TGTACAATCAA-CTTACAGAC-TT-AAACTGAT
Cap2G3a     15   AATGAGAAA-TATCTGGCTCTAAAAC--TAAAACGG-AGCTATGT-ACCAGAATGA-TTGTCAAGG-TGTATATT-AAACTTACAGAG--TTAAACAATT
Cap2G3b     16   AATGAGAAA-TATCTTGCTCTAAAAC--AAAAACGG-AGCAATGT-AACAGAATGA-TTGTCAAGG-GATACATT-AAACTTTCTGAG--TTAAGCAAGT
Cap2G4a     17   AATGAGAAA-TACCTGGCTCCAAAAC---AAAACCG-AGCTATCT-AGCAGAATGA-TTGTCAAGG-TGTATTTTCAA-CTTACAGTG--CTAAACAGAC
Cap2G4b     18   AATGAGAAA-TATCGGGCTCTAAAAC--AACAACGG-AGCTATGT-AGCAGAATGA-TTGTCAAGG-TGTATATTAAA-CTTACAGAG--TTAAACAATT
Cap2J2a     19   AAGGAGAAA-TATCTTGCTCTAAAAC--AAAAACGG-AGCTATGT-AACAGAATGA-TTGTCAAGG-TGTATATTAAA-CTTACAGAT-TTAAA-CAATT
Cap2J3a     20   AATGAGAAA-TATCTGGCTCTAAAAC--AAAAACGG-AGCTATGT-AACAGAATGA-TTGTCAAGG-TGTATATTAAA-CTTACAGAG-TTCAA-CAATT
Cap2J4a     21   AATGAGACA-TATCTTGCTCTAAAAC--AAAAACGG-AGCTATGT-AACAGAAAGA-TTGTCAAGG-TGTATATTAAA-CTTACAGAG-TTAAA-CAATT
Cap2J5a     22   AATGAGAAA-TATCTTGCTCTAAAAC--AAAAACGA-TGCTATCT-AGCAGAATGG-TTGTCTAGC-GGTACAATCTT-CCTACAGAG-TT-AAACTGAC
Cap2J5b     23   AATCAGAAA-TAGCTTGCTCTAAAAC--AAAAATGC-CGCTATCT-AGCAGAATGT-TTGTCCAGC-TGTAAAATCAT-CTTATGTAG-TT-AAATTGAT
Cap2J6a     24   AATGAGAAA-CATCTGTCTCCAAAAC--AACAACGG-AGCTATCT-AGCAGAATGA-TTGTCAAGG-TGTATATTCAT-CTTACAGAG--TTAAACAGAT
Cap2L3a     25   AATAAGAAA-TATCTGGCTCTAAAAC--AAAAGCGG-ACCTTTGT-AGCAGAATGA-ATGTCAAGG-TGTGTATTCAA-CATATAGAA--TTAAACAGAT
Cap2L4a     26   AATGACAAA-TATCTTGCTCTAAAAC--AAAAGCGC-AGCTATCT-AGCAGAATGG-TTGTCAAGC-TGAAGAATCAA-CTTACAGAG-CT-AAAGTGAT
Cap2L4b     27   AATGAGAAA-TATCTTGCTCTAAAAC--AAAAACGC-TGCTATCT-AGCAGAAAGG-GTGTCAATC-TGTACAGTCAA-CTTACAGAG-CT-AAACTGAT
Cap3A2a     28   AATCAGAAA-TATTTGGCTCTAAAAC--AAAAAAAGTAGCTATCT-AGCAGAATGA-TTGTCAAGG-TGTATATTGAA-CTTACAGAG--TTAAACAGAT
Cap3A2b     29   AATGAGAAT-CATCTGGCTCTAAAGA--ATAAGCGG-AGCTATCT-AGCAGAATGA-TTGTCAAGG-TGTATATTTAA-CTTACAGAG--TCCAACAGAT
Cap3A3a     30   AAAGAGAAA-TATGTTGGTCTAAAAC--AAAAACGC-AGCTATCT-AGCAGAATGG-TTGTTAAGC-TGTGCAATCAC-CTTACAGAG-TT-AAACTGAT
Cap3B7a     31   AATGAGAAAATATCTTGCTCTAAAAACAAAAAACGG-AGCTATGA-AACAGAATGAATTGTCAAGGGTGTATATTAAAACTTACAGAGGTTAAATCAATT
Cap3E2a     32   AATCAGAAAATATCTTGCTCTAAAAC--AAAAACGC-AGCTATCT-AGCAGAATGT-TTGTCAAGC-TGTACAATCAA-CTTACAGAG-TTCAAACTGAT
Cap3F1a     33   AATGAGAAA-TACCTTGCTCTAAAAC--AAAAACGG-AGCTATGT-AACAGAATGA-TTGTCAAGG-TGTATATTAAA-CTTACAGAG-TTAAA-CAATT
Cap3F1b     34   AATGAGAAA-TATCTTGCTCTAAAAC--AAAACCGG-AGCTATGT-AACAGAATGA-TTGTCAAGG-TGTATATTAAA-CTTACAGAG-TTAAA-CAATT
Cap3G3a     35   AATGAGAAA-TATCTTGCTCTAAAAC--AAAAGCAG-AGCTGTGC-AGCAGAATGA-TTGTCAAGG-TGTATATTAAA-ATTACAGAG--TTAAACAATT
Cap3I7a     36   AATGAGAAA-TATCTGGCTCTAAAAC--AAAAACGG-AGTTATGT-AGCAGAATGA-TTATCAAGG-TGTATATTAAA-CTAACAGAG--TTAAACAATT
Cap3I7b     37   AATGAGAAA-TATCTAGCTCTAAAAC--AAAAACGG-CGCTATGT-AGCAGAATGA-TTGTCAAGG-TGTATATTAAA-CTTACAGAG--TTAAAGAATT
Cap3J1a     38   AATGAGAAA-TATCTTGCTCTAAAGT--AAAAACGG-ACCTATGT-AACAGAATGA-TTGTCAAGG-TGTATATTTAA-CTTACAGAG-TTAAA-CAATT
Cap3J1b     39   AATGAGAAA-TATCTGGCTCTAAAAC--AAAAACGG-AGCTATGT-AACAGAATGA-TTGTCAAGG-TGTATATTAAA-CTTACAGAG-TTAAA-CAATT
Cap3J6a     40   AATGAGAAA-TATCTTGCTCTAAAAC--AAAAACGT-AGCTATGT-AACAGAATGA-TTGTCAAGG-TGTATATTAAA-CTTACATAG-TTAAA-CAATT
Cap3J6b     41   AATGAGAAA-TATCTTGCTCTAAAAC--AAAAACGG-AGCTATGT-AACAGAATGA-TTGTCAAGG-TGTATATTAAA-CTTACAGAG-TTAAA-CAATT

                 .........+.........+.........+.........+.........+.........+.........+.........+.........+.........2
            Con  GTGTGAA-TGTAGCAGTTCAGA-AACCCTTTCTTT-GGA--GAATCTGGAAAGAGA--TATTTCCAGCCATATACAGACTCTTAAGAATTAGCTTTAA-G
            01   ACGTGAA-TGTAGCAGTTCAGA-AAACATTTCTTT--GA--GAATCTGGAAAGAGA--TATTTCCAGCCATATACAGACACTTAAGAATTAGCTTTAC-G
            02   GTGTGAA-TGTAGCAGTTGAAA-AACCCTTTCTTT-GGA--GAATCTGGAAAGAGA--TATTTCCCGCCATATACAGGCTCCTAAGAATTAGCGGTAA-T
            03   ATGTGAA-TGTAGCAGTTCAGA-AAACATTTCTTT--GA--GAATCTGAAAAGAGA--TATTTACAGCCATATACAGACACTTAAGAATTAGCTTTAC-G
            04   GCGTGAA-TGTAGCAGTTCACT-AACCGTTTCTTT-AGA--GAATCTGGAAAGAGA--TATTTCCACCCATACTCAGACTCTTAAGAATTAGCTTTAT-G
            05   GTGTGTT-T-CAGCAGTTCAGAAA-TCCTTTGCTT-C-A--GAATCTGGAAACAGA--TATTTACCGCCATCTACAGGCATATGAGACCTACCAGTAA-G
            06   GTGTGTG-TTCAGAGGTTCTGAAA-TCTTTTCTTT-GGG--GAATCTGGAAACAGA--TATTTCCAGACTTGCAGCGGCATTTAATACCTTGCAGTAA-G
            07   GTGTGAA-TGTAGCAGTTCACT-AATCCTTTCTTT-GGA--GAATCTGGAAAGAGA--TATTTCCAGCTTTATGCAGACTCTTAAGAGTTAGCTTTAA-G
            08   GTTTGAG-TGTAGCAGTTCACC-AACCCTTTCTTT-GGA--GAATCTGGAAAGAGA--TATTTACAGCCATATACAGACTCTTAAGAATTAGCTTTAA-G
            09   ACGTGAA-TGTAGCAGTTCAGA-AAACATTTCTTT--GA--GAATCTGGAAAGAGA--TATTTCCAGCCATATACAGACACTTAAGAATTAGCTTTAC-G
            10   ATGTGAA-TGTAGCAGTTCAGA-AAACATTTCTTT--GA--GAATCTGAAAAGAGA--TATTTACAGCCATATACAGACACTTAAGAATTAGCTTTAC-G
            11   GTGTGTT-T-CAGCAGTTCAGAAA-TCCTTTGCTT-C-A--GAATCTGGAAACAGA--TATTTACCGCCATCTACAGGCATATGAGACCTACCAGTAA-G
            12   CTATGGAATGTAGCAGTTATCCCAACCCTTTTTTTGGAAGGAATTGGGAAAGAAAA-TTTTCCCAGCCGCTTACAGATTCTTAAGAAATTACTTTAAAG
            13   GTGTG-AATGTATCAGTTCACTAAGCC--TTTCTTTGGA--GAATCTGGAAAGAGA-TTATTTCCAGCCGCTTACAGACTCTTAAGAATTAGCTTTAA-G
            14   GTGTGTG-TGCAGCATTTCTGAAACTCGTTTCTTT-GGA--GAATCTGTAAACAGA--TATTTCCTGCCATATACAGGCAAAGAAGAACTGGCAGTGA-G
            15   GTGTG-AATGTAGCAGTTCACTAAGCT--TTTCTTTTTA--GAATCTGGAAAGAGA--TATTTCCAGCCGCTTACAGACTCTTAAGAATTAGCTTTAA-G
            16   ATGTG-AATGTAGCAGTTCAATAACCC--TTTCTTTGGA--GACTCTGG----AGA--CATTTCCAGCTTTATACAGACTCTTAAGAATTAGCTTTAA-G
            17   GTGTGAA-TGTAGCAGTTCAGA-AACCCTTATTTT---A--GAATCTTGAAAGAGA--CATTTCCAGCCATATACAGATATATAAGAATGAACGGTAA-G
            18   GTGTCAA-TGTAGCAGTTCAGT-CACCCTTTCTTT-GGA--GAATCTGGAAAGAGA--TATTTCCAGCCATATACAGGCTCCTAAGAATTAGCTTTAA-G
            19   GTGTGAA-TGTAGCAGTTCACC-AACCCTTTCTTT-GGG--TAATCTGGAAAGAGA--TATTTCCAGCTTTATACAGACTCTTAAGAATTAGCTATAA-G
            20   GTTTGAA-TGTAGCAGTTCACC-AACCCTTTCTTTTGGA--GAATCTGGAAAGAGA--TATTTACAGCCATATACAGACTCTTAAGAATTAGCTTTAA-G
            21   GTGTGAA-TGTAGCAGTTCACC-AACCCTTTCTTT-GGG--GAATCTGGAAGGAGA--TATTTCCAGCTTTATACAGACTCTTAAGAATTAGCTTTAA-G
            22   GTGTCTG-TGCAGTAGCTCAGAAA-TCCTCTCTTT-GGA--GAATCTGGAAATAGA--TATTTCCAGCCATATACAGGCTTATGAGAACTAGCAGTAA-G
            23   GTGTGTG-TGCAGAGGTTCTGAAA-TCTTTTCTTT-GGG--GAATCTGGAAACAGA--TATTTCCTGCCTTACACAGGCATTTAAGTCCTAGCAGTAA-G
            24   GTGTGTA-TATAGCAGTGCCAA-AACGCTTTCTTT-TGA--TAATCTAGAAAGAGA--TATTTCCAGCCACATACAGGAACATAAGAACTGGCAGAAA-G
            25   GTGTGAA-GGTAGCAGTTCAGA-AACCCTTTCTTT-TGA--GAACCTAGAAAGAGA--TATTTCCAGCCTTATACATGCACAAAAGA-TTAGTGGTAA-C
            26   GTGTGTG-TGCAGCAGTTCAGAAA-TCGTTTCTTT-GGA--GAATCTGGAAACAGA--TATTTCCAGCCTTATACAGGCATAGAAGAACTTCCAGTAA-G
            27   GTGTGTG-TGCAGCAGTTCAGAAA-TCGTTTCTTT-GGA--GAATCTGGAAACAGA--TATTTCCAGCCATATACAGGCATATAAGAACTGGCAGTAA-G
            28   ATGTGAA-TGTAGCAGTTCAGA-AACCCTTTCTTT-GGA--GTATCAAGAAAGTGA--TACTTCCAGCCATATACAGGCACATAAGAATTAGTGGTAA-G
            29   ATGTGAA-GGTAGCAGTTCAGA-AACCCATT-TTT-GGA--GAAGGTAGAAAGAGA--TATTTCCAGCCTTATACAGCCACATA---------------
            30   GTGTAAG-TGCAGCAGTTCAGAAG-TCCTTTCTTT-GCA--CAATAGGGAAGGAGA--TATTTCCAGACGTATACAGGCACATAAGAACTAGCAGTAA-G
            31   GTGTGAAATATAGCAGTTCACC-AACCCTTTCTTT-GGA--GAATCTGGAAAGAGA--TATTTCCAGCTTTATACAGACTCTTAAGAATTAGCTTTAA-G
            32   GTGTGTG-TGCAGCAGTTCAGAAA-TCTTTTCTTT-GGA--GAATCTTGAAACAGA--GATTTCCAGGAATATACAGGCCTATAAGAACTAGCAGTAA-G
            33   GTGTGAA-TGTAGCAGTTCACC-AACCCTTTCTTT-GGG--GAATCTGGAAAGAGA--TATTTCCAGCTTTATACAGACTCTTAAGAATTAGCTTTAA-G
            34   GTGTGAA-TGTAGCAGTTCACC-AACCCTTTCTTT-GGC--GAATCTGGAAAGAGA--TATTTCCAGCTTTATACAGACTCTTAACAATTAGCTTTAA-G
            35   GTGTGAA-TGTGGCAGTTCACT-AACCCTTTCTTT-GCA--GAATCTGGAA-GAGA--TATTTCCAACCATATACAGACTCTTAAGAATTAGCTTTAA-G
            36   GTGTGAA-TGCAGCAGTTCACT-AACCCTTTCTTT-GGA--GAATCTGGAAAGAGA--TATTTCCAGCCATATACACACTCTTAATAATTTGCTTTAA-G
            37   CTGTGAA-TGTAGCAGTTCACT-AACCCTTTCTTT-GGA--GAATCTGGAAAGTGA--AATTTCCAGGAATATACAGACTCTTAAGAAGTAGCTTTAC-G
            38   GCGTGAA-TGTAGCAGTTCACC-AACCCTTTCTTT-GAA--GAATCTGGAAAGAGA--TATTTACAGCTTCATTCAGACTCTTAAGAATTAGCTTTAA-G
            39   GTGTGAA-TATAGCAGTTCACC-AACCCTTTCTTT-GGA--GAATCAGGAAAGAGA--TATTTACAGCTTTATACAGACTCTTAAGAATTAGCTTTAA-G
            40   GTTTGAA-TGTAGCAGTTCAGC-AACCCTTTCTTT-GGA--GAATCTGGAAAGATA--TATTTACAGCCATATACAGACTCTTAAGAATTAGCTTTAA-G
            41   GTTTGAA-TGTAGCAGTTCACC-AACCCTTTCTTT-GGA--GATTCTGGAAAGAGA--TATTTACAGCCATATACAGACTCTGAAGAATTAGCTTTAA-G
```

Figure S1, page 1 of 12

Cap (n=41), 2 of 2

```
    .........+.........+.........+.........+.........+.........+.........+.........+.........+.........3
Con ATCGCATCT--AAATCATA?GAGAAACGTCTCTTTC-AAACTGCTCCACTGTGTG--TTCGTTCACCTT?-GGAAGTTGCATCTATGTTT-AGATTCAGC
01  ATCGCATCT--AAACCATACGAGAAACGTCTCTTTC-AAAGTGCTCTGCTGTGTG--TTCGTTGACCTTAGGAAGTTTGCATCTATGTTT-AGATTCAGC
02  GTCGCGTCT--GAACCACAGGAGAATCGTCTCTTTC-AAACTGCTCTCTTATGTG--TTCGTTCACCTTC-GGATGTTGCATCTATGTTT-AGATTCAGC
03  ATCGCATCT--AAACCATACGAGAAACGTCTCTTTC-AAACTGCTCTGCTGTGTG--TTCGTTGACCTTAGGAAGTT-GCATCTATGTTT-AGATTCAGC
04  ATCGCACCT--AATTCATACGAGAAACGTCTCTTTC-AAACTGCTCCACTGTGTG-AT-CGTTCACCTTCGGAAGTT-GCATCTATGTTT-AGATTCTGC
05  ACTGCGTTT--AAAACCCAGGAGAAAAGACTAGTTC-AAACTGCTTTGCTTTGTG--TTCGTTCACCTTA-GGAAGTTCCATGTATGTTT-AGATTCAGC
06  ACTGCGTTT--AAAACACAGGAGAAACGTCTAGTTC-AAACTACTCTGCTTTGTG--TTCCTTCATCTTA-GGAAGTTGCATCTATGTTTTAGATTCAGC
07  ATCGCATCT--AAATTATAAAAGAAACGTCTCTTTC-AAACTGCTCCACTGTGTG--TTCGTTCACCTTC-GGAAGTTGCATCTATGTTT-AGATTCTGC
08  ATCGCATCT--AAATCATACGAGAAACGTCTCTTTC-AAACTGCTCCACTGTGTG--TTGGTTCACCTTCTGAAA-TTGCATCTATGTTT-AGATTCTGC
09  ATCGCATCT--AAACCATACGAGAAACGTCTCTTTC-AAAGTGCTCTGCTGTGTG--TTCGTTGACCTTAGGAAGTT-GCATCTATGTTT-AGATTCAGC
10  ATCGCATCT--AAACCATACGAGAAACGTCTCTTTC-AAACTGCTCTGCTGTGTG--TTCGTTGACCTTAGGAAGTT-GCATCTATGTTT-AGATTCAGC
11  ACTGCGTTT--AAAACCCAGGAGAAAAGACTAGTTC-AAACTGCTTTGCTTTGTG--TTCGTTCACCTTA-GGAAGTTCCATGTATGTTT-AGATTCAGC
12  ATCGCCTTCTAAAATGATACGAGAAACGTTTTTTTCCAAACTGTTCAATTGGGGGGTTTGTTTCCCCTTTCGGAAAGTGCAAACAAGTTC-AGATTTTGG
13  ATAGCATCT--AAATGATAAGAGAAACGTCTCTTTC-AAACTGCTCCACTGTGTG--TTGGTTCACCTT-CGGAAGTTGCAACTATGTTT-AGATTCTGC
14  ACTGCGTTT--AAAAAACAGGAGAAACGTCTATTTC-AAACTGGTCTGTTTTGTG--TTCGTTCACCTTA-GGAAGTTGCATCT--GTTT-AGATTCAGC
15  ATCGCATCT--AAATGATACGAGAAACGTCTCTTTC-AAACTGCTCCACTGTGTG--CTCGTTCACCTT-TGGAAGTTGCATCTATGTTT-AGATTTT-C
16  ATCGCATCT--AAATGATACGAGAAACGTCTCTTTC-AAACTGCTCCACTGTGTG--CTCGTTCACCTT-TGGAAGTTGCATCTATGTTT-AGATTTTTC
17  GCCGTGTCT--ACACCACAGGGGAGACGTCTCTTTC-ATACTGCTCGGCTGTGTG--TTCGTTCACCTTC-GGAAGTTGCATCTATGTTT-ACATTCAGT
18  ATCACATCT--AAATCACAGGAGAAACGTCTCTTTC-AAACTGCTCCGCTGTGTG--TTCGTTCGCCTTC-GGAAGTTGCATCTATGTTT-AGATTCAGC
19  ATCGCATCT--AAATGATACGAGAAAAGTCTCTTTC-AAACTGCTCCACTGTGTG--TCCGTTCAGCTTC-GGAAGTTGCGTCTATGTTT-AGATTCTGC
20  ATCGCATCT--AAATCATACGAGAAACGTCTCTTTC-AAACTGCTCCACTGTGTG--TTGGTTCACCTTTCGGAAGTTGCATCTATGTTT-AGATTCTGC
21  ATCGCATCT--AAATGATACGAGAAAAGTCTCTTTC-AAACTGCTCCACTGTGTG--TTCGTTCACCTTC-GGAAGTTGCATCTATGTTT-AGAGTCTGC
22  ACTCCGTTT--AAAACACAGGAAAAATGTCTAGTTC-AAACTCTTCTGCTTTGTG--TTCGTTCACCTTA-GGAAGTTGCATCTATGTTT-AGATTCAGT
23  ACTGCTTTT--AAAATACAGCAGAAACGTCTAGTTC-AAACTGCTCTGCTTTGTG--TTCGTTCACCTTA-CGAACTTGCATCTATGTTT-AGATTCAGC
24  ATCGTGTCT--AAACCACAGGAGAAACGTCTCTTTC-AAACTGCTCTGCTGTGTG--TTCGTTCACCTTA-GGAAGTTGCATCTATGTTT-AGATTTAGA
25  ATCGCGTCT--AAACCACAGGAGAAACGTCCCTTTC-AAACTGCTCTGCTGTATG--TTCGTTCACCTAA-GGAAGTTGCATCTATGTTA-A-ATTCAGC
26  ACTGCATTT--AAAACACAGGAGAAACGTCTATTTC-AAACTGCTCTGCTTTGTG--TTCGTTCACCTTA-GGAAGTTGCCTCTATGTTT-AGATTCAGC
27  ACTGCGTTT--TAAACACAGGAGAAACATCTCGTTC-AAACTGCTCTGCTTTGTG--TTCGTTCACCTTA-GGTAGGTGGCCCTATGTTC-AGAATGAGC
28  ATCACGTCT--AA-CCACAGGAGAATCCTCTCTATC-AAACTGCTCTGCTCTGTG--TTCGTTCACCTTA-AGAAGTTGCATCTACATTA-AGATTCAGC
29  ------TAT--AAAGCACAGGAGAAACGTGTCTTTC-AAACTGCTCTGCAGTGTG--TTCGTTCACCTTA-GAAAGTTGCAACTACGTTT-AGATTCAGC
30  ACTGCGTTT--AAAACACGGGAGAAACGACTAGTTC-AAACTGCTCTGCTTTGTG--TTCGCTCACCTTC-GGAAGTTGCATTTATGTTT-AGATTCAGC
31  ATCGCATCT--AAATGATACGAGAAATGTCTCTTTC-AAACTGCTCCACTGTGTG--TTCGTTCACCTTC-GAAAGTTGCATCTATGTTT-AGATTCTGC
32  ACTGTGTTT--AAAACACAGGAGAAACGAGTTGTTC-AAACTGCTCTACTTTGTG--TTCGTTCACCTTA-GGAAGTTGCATCTATGTTT-AGATTCAGT
33  ATCGCATTT--AAATGATACGAGAAAAGTCTCTTTC-AAACTGCTCCACTGTGTG--TTCGTTCACCTTC-GGAAGTTGCATCTATGTTT-AGATTCTGC
34  ATCGCATTT--AAATGATATGAGAAAAGTCTCTTTC-AAACTGATTCACTGTGTG--TTCGTTCACCTTC-GGAAGTTGCATCTATGTTT-AGATTCTGC
35  ATCGCATCT--AAATCATACGAGAACCGTCTCTTTC-AAACTGCTCCAATGTGTG--TTCGTTCACCATA-GGAAGTTGCATCTATGTTT-AGATTCTGG
36  ATCGCATCT--AAATCACATGAGAAATGTCTCTTTC-AAACTGCTCCACTGTGTG-TT-CGTTCAACTTCGGAAGTT-GCATCTATGTTG-AGATTCTGC
37  ATCGCATCT--AAATCATACGAGAAACGTCGCTTTC-AAACTGCTCCACTGTGTG-TTTCGTTCACCTTCGGAAGTTTGCATCTATGTTT-AGATTCTGC
38  ATCGCATCT--AAATGATACGAGAAACGTCTCTTTT-AAACTGCTCCACTGTGTG--TTGGTTCACCTTC-GGAAGTTGCATCTATGTTT-AGATTCTGC
39  AACGCATCT--AAATGATACGAGAAACGTCTCTTTC-AAACTGCTCCACTGTGTG--TTGGTTCACCTTC-GGAAGTTGCATCTATGTTT-AGATTCTGC
40  ATCGCATCT--AAATCATACGAGAAACGTCTCTTTC-AAACTGCTCCACTGTGTG--TTGGTTCACCTTC-GGAAGTTGCATCTATGTTT-AGATTCTGC
41  ATCGCATCT--AAATCATACGAGAAACGTCTCTTTC-AAACTGCTCCACTGTGTG--TTGGTTCACCTAC-GGAAGTTGCATCTATGTAT-AGATTCTGC

    .........+.........+.........+.........+.........+.........+.........+.........+.........+.........4
Con GAGTTAGAAACACGTTTCT?C?A-AAAGCT-GCGTTT-GGATATTCC-TGAGTGAAT-GGGAGT-ATAGGGTTT-
01  GAATTAGAAACACGTTTCTTCTG-AAAATTTCCGTTT-GGATATTCCTGAAGTGAAT-GGGAGT-ATAGGGTGA-
02  GAGTTAGAAACACGTTTCTTCAA-AAAGCT-GCGTTT-GGATATTCT-TGAGAGAAT-GGGAATAATGGGAGT--
03  GAGTTAGAAACACGTTTCTTCTG-AAA-TTTCCGTTT-GGATATTCCTGA-GTGAAT-GGGAGT-ATAGGGTGT-
04  GAGTTAGAAACACGTTTCTCCAA-AAAGCT-GCGTTT-GGATATTCC-TGAGTGAAT-GGGAGT-ATAAAGGTTT
05  GAGTTAGTAACACGTTTCTTCTA-AAAGCA-ACGTTT-GGATATTCC-TGTGAGAAA-GAGCTT-GTAGGCCTT-
06  GAGTTGGAGACACGTTTCTTCTA-AAACCT-GCGTTT-GGATATTCC-ACAATGTCT-GAGAAT-GAAGGCCGT-
07  GAGTTAGAAAGACGTTTCTCCAA-AAAGCT-GCGTTT-GGATATTCC-TGAGTGAAT-GGGAGT-ATAGTGTTT-
08  GAGTTAGAAAGACGTATCTCCAA-AAAGCT-GCGTTT-GGATATTCC-TGAGTGAAT-GGGAGT-ATAGGGTTT-
09  GAATTAGAAACACGTTTCTTCTG-AAA-TTTCCGTTT-GGATATTCCTGA-GTGAAT-GGGAGT-ATAGGGTGT-
10  GAGTTAGAAACACGTTTCTTCTG-AAA-TTTCCGTTT-GGATATTCCTGA-GTGAAT-GGGAGT-ATAGGGTGT-
11  GAGTTAGTAACACGTTTCTTCTA-AAAGCA-ACGTTT-GGATATTCC-TGTGAGAAA-GAGCTT-GTAGGCCTTT
12  ATTTAGAAAAAACGTTTTTTCCA-AAAAGC-GCGTTA-AGATATTCC-TAATTCAAT-GGGGGT-ACAGGGTTT-
13  GAGTTAGAAACACGTTTCTCCAA-AAAGCT-GCGTTT-GGATATTCC-TGAGTGAAT-GGGAGT-ATAGGGTTT-
14  GAGTTAGAAACAAGTTTCTTCTA-AAAGCT-TCGTTT-TGATATTCT-AGAGTGAAT-GGGAGT-ATAGGCTGT-
15  GAGTTAGAAAGACGTTACTCAAA-AAAGCT-GCGTTT-GGATATTCC-TGAGTGAAT-GGGAGT-ATAGGGTTT-
16  GAGTTAGAAAGACGTTACTCAAA-AAAGCT-GCGTTT-GGATATTCC-TGAGTGAAT-GGGAGT-ATAGGGTTT-
17  GAGTTAGAAACACGTTTCTCCAA-AAAGCG-GCGTTT-GGATATTCC-TGAGTAAAT-GGGAGT-ATATGGTGT-
18  GAGTTAGAAACACGTTTCTCCTG-AAAGCT-GCGTTT-GGATATTCC-TGAGTGAAT-GCGAATAATGGGTGT--
19  GAGTTAGAAAGACGTTTCTCCTA-AAAGCT-GCGTTT-GGATATTCC-TGAGTGAAT-GGGAGT-ATAGGGTTT-
20  GAGTTAGAAAGACGTTTCTCCAATAAAGCT-GCGTTT-GGATATTCC-TGAGTGAATTGGGAGTTATAGGGTTT-
21  GAGTTAGAAAGACGTTTCTCCAA-AAAGCT-GCGTTA-GGATATTCC-TGAGTGAAT-GGGAGT-ATAGGGTTT-
22  GAGTTATAAACACGTTTCTTCTA-AAAGCA-GCGTTT-GGGTATTCC-AGTGTGAAA-GAGCCT-GCAGGCCGT-
23  GAGTTAGAAACACGTTTCTTCTA-AAGCA--GTGTTT-GGATATTCT-AGTGTGAAA-GAGTCT-GTAGGCCGT-
24  GAGTTAAAAACACGTTTCTTCTA-AAAGAT-GCCTTT-GGATATTCC-TGAGTGAAT--GGGATCATAGGGTGT-
25  GAGTTAGAAACACGTTTCTTCTA-AAAGCT-G--TTT-CCATATTCC-TCCATGAAG-AGGAGT-GTAGCGTGT-
26  GAGTTAGAAACACCTTTCTTTTG-TAATCT-GCGTTT-GGATATTCC-AGAGTGAAT-TACACT-ATACGATGT-
27  GAGTTAGAAACACGTTTCTTCTA-GTAGCT-GCGTTT-GGATATGCC-AGAGTGATT-GAAACT-ATAGGCTGT-
28  GACTTAGAAACACGTTCTTTCTG-AAAGCT-GCGTTT-GTATATTCC-TGAGTGAAT-GGGATT-ATACCGTGT-
29  GTGTTAGAGACACGTTTCTTCTA-AAAGCT-GTGTTT-GGATATTCC-TGAGTGAAT-GTGACT-ATAGGGTGT-
30  GAATTAGAAAAACGTTTCTTCTC-AAAGCT-GCCTTT-GGATATTAC-AGAGTGATT-TAGACT-ATAGGCTGT-
31  GAGTTAGAAAGACGTTTCTCCAA-AAAGCT-CTGTTT-GTATATTCC-TGAGTGAAT-GGGAAT-ATAGGGTTT-
32  GAGTTACAAACAGCTTTCTTCTA-AAAGCT-GCGTTT-GGATATTCC-AGAGTGAAT-GAGGCC-ATAGGCTGT-
33  GAGTTAGAAAGACGTTTCTCCAA-AAAGCT-GCGTTT-GGATATTCC-TGAGTGAAT-GGGAGT-ATAGGGTTT-
34  GAGTTAGAAAGACCTTTCTCCAA-AAAGCT-GCGTTT-GGATATTCC-TGAGTGAAT-GGCAGT-ATAGGGTTT-
35  GAGTTAGAAACACGTTTCTCCAA-AAAGCT-GAGTTT-GGATATTCC-TGAGTGAAT-GGGAGT-ATAGGGTGT-
36  GAGCTGGAAACACGTTTATCCAA-AAAGCT-GCGTTT-GGATATTCC-TGAGTGAAT-GGGAGT-ATAGGGTTT-
37  GAGTTAGAAACACGTTTCTGCAA-AAAGCTTGCGTTTTGGATATCCCCTGAGTGAAT-GGGAGT-ATAGGGTTT-
38  GAGTTAGAAAGACGTTTCTCCAA-AAAGCT-GCGTTT-GGATATTCC-TGAGTGAAT-GGGAGT-ATAGGGTTT-
39  GAGTTAGAAAGACGTTTCTCCAA-AAAGCT-GCGATT-GGATATTCC-TGAGTGAAT-GGGAGT-ATAGGGTTT-
40  GAGTTAGAAAGACGTTTCTCCAA-AAAGCT-GCGTTT-GGATATTCC-TGAGTGAAT-GGGAGT-ATAGGGTTT-
41  GAGTTAGAAAGACGTTTCTCCAA-AAAGCT-GCGTTT-GGATATTCC-TGAGTGAAT-GGTAGT-ATAGGGTTT-
```

Figure S1, page 2 of 12

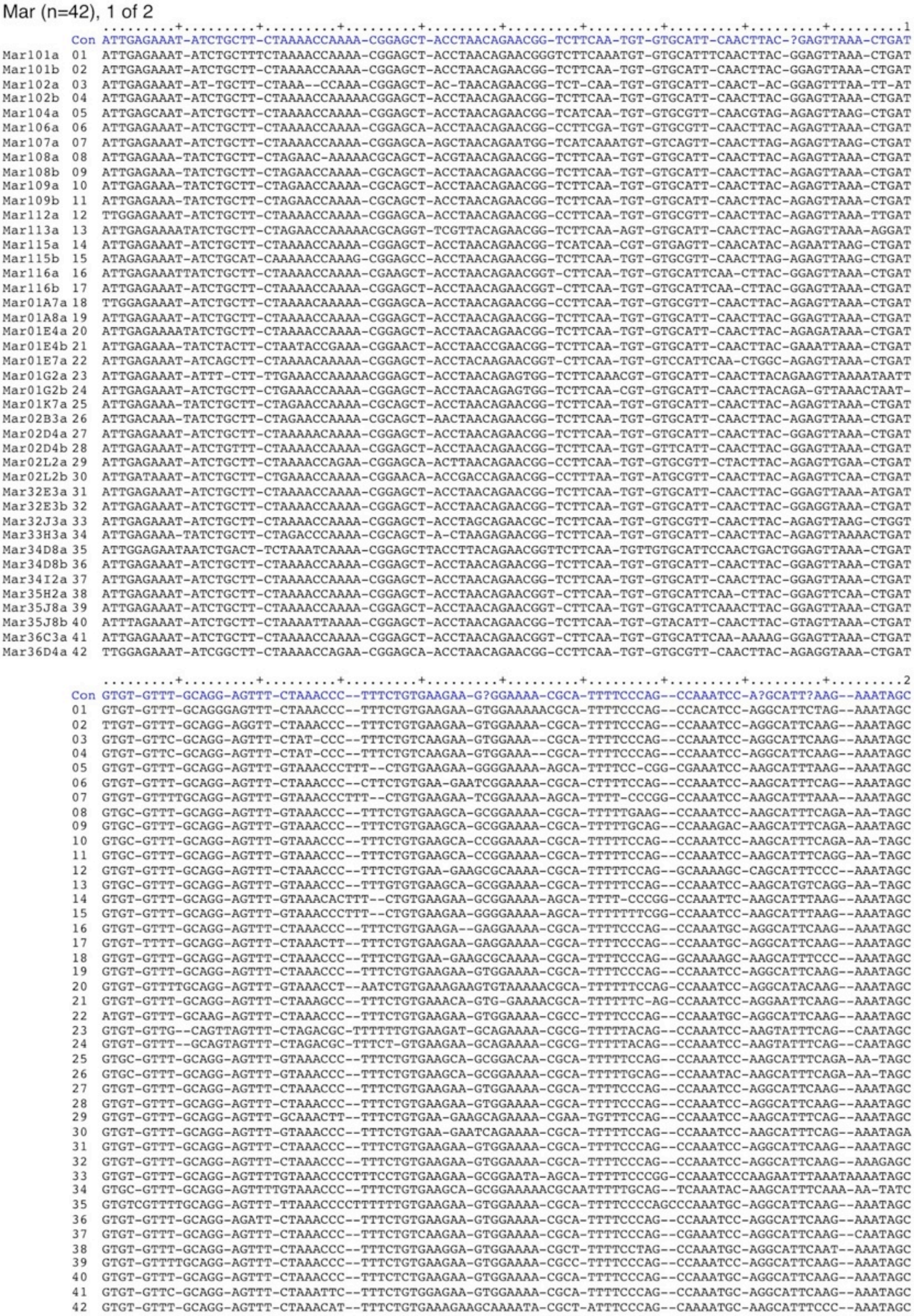

Figure S1, page 3 of 12

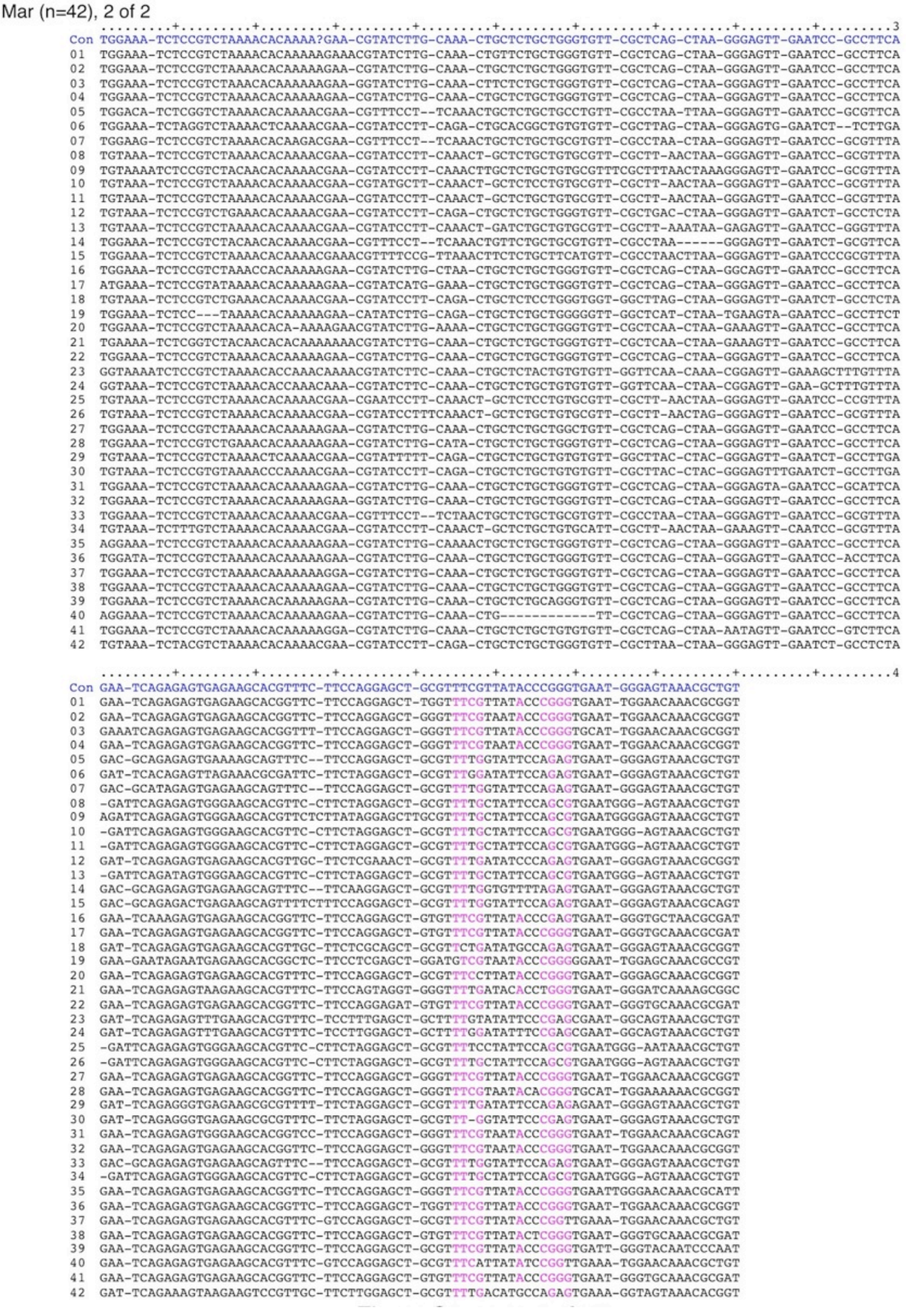

Figure S1, page 4 of 12

Owl (n=40), 1 of 2

```
                 .........+.........+.........+.........+.........+.........+.........+.........+.........+.........1
             Con ACAGAGAAATATCTGCTTCTAAAACC-AAAACGGA-GCTATCTAA-CTGAATGGTTGTCAATGTGTGCATTCAACTGACAGAGTTAAACTCACGTGTGGA
Owl022a  01  ACAGAGAAATATCTGCTTCTAAAACC-AAAACGGA-GCTATCTAA-CTGAATGGTTGTCAATGTGTGCATTGAACTGACAGAGTTAAACTCACGTGTGGA
Owl036a  02  GCAGAGAAATACCTGCTTCTAAAACC-AAAACGCA-GCTATCTAA-CTGAATGACTGTCAATTTGTGCATTCAACTGACAGAGTTAAACTCATGTGTGGA
Owl054a  03  ACAGAGAAATATCTGCTTCTGAAAAC-AAAACGGA-GCTATCTAA-CTGAATGGTTGTCAATGTGTGCATTCAACGCACAGAGTTAAATTCACTTGTGGA
Owl203a  04  ACAGAGAATTATCTGCTTCTAAAACC-AAAACGGA-GCTATCTAA-CTGAATGGTTTTCCATGTGTGCATTCAACTGACAGAGTTAAACTCATGTGTGGA
Owl301a  05  ACAGAGAAATATCTGCTTCTAAAACC-AAAATAGA-GCTATCTAA-CTGTATGGGTGTCCATGCGTGCATTCAAATGACAGGGTTAAACTCATGTGTGGT
Owl301b  06  ACAGAGAATTATCTGCTTCTAAAACC-AAAACGGA-GCTATCTAA-CTGAATGGTTTTCCATGTGTGCATTCAACTGACAGAGCTAAACACATGTGTGGA
Owl302a  07  ACAGAGAAATATCTGCTTCTAAAACC-AAAACGGA-GCTATCTAA-CTGAATGGTTGTCAACGTGTGCATTGAACTGACAGAGTTAAACTCACGTGTGGA
Owl303a  08  ACAGAGTAATATCTGCTTTTAAATCC-AAAACGGA-GCTACCTAA-CTGAATGGTTGTCAATGTGTGCATTGAACTGACAGAGTTAAACTCACGTGTGAA
Owl304a  09  ACAGAGAAATTTCTGCTTCTAAACCC-AAACGGGA-GTTATCTAA-CGGAAGGGTTGCCAATGTGTGCATTCAACGGACAGAGTTAAATTCAGGTGTGGA
Owl305a  10  ACAGGGAAATATCGGCTTCTAAAACC-AAAACGGA-GCTATCTAA-CTGAATGGTTGTCAATGTCTGCATTCAACTGAGAGAGTTAAACTCACGTGTGGA
Owl306a  11  ACAGAGAAATATCTGCTTCTAAAACC-AAAACGGA-GCTATCTAATCTGAATGGTTGTCAATGTGTGCATTCAACTGACAGAGTTAAACTCATATGTGGA
Owl306b  12  ACAGAGAAATATCTGCTTCTGAACCC-AAAACGGA-TCTATCTAA-CTGAATGGCAGTCAATTTGGGCATTCAACTGACAGAGGTAAACTCATGTGTGTA
Owl307a  13  ACAGAGGAATATCTGCTTCTAAAACC-AAAACGGA-GCTATCTGA-CTGAATGGTTGTCAATGAGTGCATTCAACTGACGGAGTTAAACTCAGGGGTGGA
Owl308a  14  ACAGAGAAATATCTGCTTCTAAAACC-AAAACGGA-GCCATCTAA-ATGAATGGTTGTCAACGTGTGCATTTAACTGACGGAGTTAAACTCACGTGTGGA
Owl403a  15  ACAGAGAAATATCTGCTTCTAAAACC-AAAACGGA-GCTATCTAA-CTGAATGGTTGTCAATGTGTGCATTCACCTGACAGAGTTAAACTCACGCGTGGA
Owl404a  16  ACAGAGAAATATCTGCTTCTAAAAACCAAAACGGA-GCTATCTAA-CAGAATGGTTGTCAGTGTGTGCATTGAACTGACAGAGTTAAACTCACGTGTGGA
Owl405a  17  ACAGAGAAATATCTGCTTCTAAAATC-AAAAGGTT-GCTATCTAA-CTGAATGGTTGTCAATGTGTGCATTCAACTGACAGAGTTAAACTCAGGTTTGGT
Owl407a  18  ACAGAGAAATATCTGCTTCTAAAACC-AAAACGGA-GCTATCTAA-CAGAATGGTTGTCCATGTGTGCATTCAACTGACAGAGTTAAACTCACGTGTGGA
Owl407b  19  ACAGAGAAATATCTGCTTCTAAAACC-AAGACGGA-GCTATCTAA-CTGAATGGTTGTCCATGTGTGCATTCAACTGACAGAGTTAAACTCACGTGTGGA
Owl410a  20  ACAGAGAAATATCTGCTTCTAAAACC-AAAACGGA-GCTATCTAA-CTGAATGGTTGTCAATGTGTGCATTGAACTGACAGAGTTAAACTCACGTGTGGA
Owl410b  21  ACAGAGAAATATCTGCTTCTAAAACC-AAAACGGA-GCTATCTAA-CTGAATGGTTGTCAATGTGTGCATTGAACTGACAGAGTTAAACTCACGTGTGGA
Owl411a  22  ACAGAGAAATATCTGCTTCTAAAACC-AAAACGGA-GCTATCGAA-CTGAATGGTTGTCAATGTGTGCATTGAACTGACAGAGTTAACCTCACGTGTGGA
Owl411b  23  ACAGAGAAATATCTGCTTCTAAAACC-AAAACGGA-GCTATCTAA-CTGAATGGTTGTCAATGTGTACATTGAACTAACAGAGTTAAACTCACGTGTGGA
Owl413a  24  ACAGAGAAATATCTGCTTCTAAAACC-AAAACGGA-GCTATCTAA-CTGAATGGTTGTCAATGTGTGCATTGAATTGACAGAATTAAACTCACGTGTGGA
Owl413b  25  ACAGAGAAATATCTGCTTCTAAAACC-AAAACGGA-GCTATCTAA-CTGAATGGTTGTCAATGTGTGCATTGAACTGACAGAGTTAAACTCACGTGTGCA
Owl414a  26  ACAGAGAAATATCTGCTTATAAATCC-AAAACGGA-GCTACCTAA-CTGAATGGTTGTCAATGTGTGCATTGAACTGACACAGTTAAACTCACGTGTGGA
Owl414b  27  ACAGAGAAATATCTGCTTCTAAAAGC-AAAACGGA-GCTATCTAA-CTGAATGGTTGTCAATGTGTGCATTGAACTGACAGAGTTAAACTCACGTGTGGA
Owl419a  28  ACAGAGAATTATCTGCTCTTAAA-CC-AAAACGGA-GCTATCTAA-CTGAATGGTTGTCAATGTCTGCATTCAACTGAGAGAGTTAAACTCACGTGTGGA
Owl419b  29  ACAGAGAAATATCTGCTCCTAAAACC-AAAACGGA-ACTATCTAA-CTGAATGGTTGTCAATGTGTGCATTCAACTAACAGAGTTAAACTCACGTGTGGA
Owl423a  30  ACAGAGAAATACCTGCTTATAAAACC-AAAACGGA-GCTATCTAA-CTGAATGGTTGTCAAAGTGTGCATTGAACTGACAGAGTTAAACTCACGTGTGGA
Owl423b  31  ACAGAGAAATATCTGCTTCTAAAACC-AAA-CGGA-GCTATCTAA-CTGAATGGTTGTCAATGTGTGCATTGAACTGACAGAGTTAAACTCACGTGTGGA
Owl425a  32  ACAGAGAAATATCTGCTTCTAAAACC-AAAACGGA-GCTATCTAA-CTGAATTGTTGTCCATGTGTGCATTCAACTGATAGAGTTAAACTCAAGTGTGGA
Owl425b  33  ACAGAGAAATATCTGCTTCTAAAACC-AAAACAGA-GCTATCTAA-CTGTATGGTTGTCCGTGTGTGCATACAACAGACAGATTTAACTTATGTGTGGA
Owl426a  34  ACAGAGAAATATCTCCTTCGAAAACC-AAAACGGA-ACTATCTAA-CGGAATGGTTGTCAATGTGTGCATTCAACTGACAGAGTTAAACTCACGTGTGGA
Owl426b  35  ACAGAGAAATATCTGCTTCGAAAACC-AAAACGGA-GCTATGTAA-CGGAATGGTTGTCAATGTGTGCATTCAACTGACAGAGTTAAACTCACGTGTGGA
Owl432a  36  CTATAGAAATATCTGCTTCTACAACC-AAAACGGA-GCTATCTAA-CTGAATGGTTGTCAATGTGTGCATTCAACTGACAGAGTTAAAATCACGTGTGGA
Owl432b  37  ACAGAGAAATATCTGCTTCTAAAACC-AAAACGGA-GCTATCTAA-CTGAATGGTTGTCAATGTGTGCTTTCACCTGACAGAGTTAAACTCACGTGTGGA
Owl436a  38  ACAGATAAATATCTGCTTCTAAAACC-AAAACGGA-GCTATCTAA-CTGAATGGTTGTCAATGTGTGCATTGAACTGACAGAGGTAAACTCACGTGTGGA
Owl438a  39  ACAGAGAA-TATCTGCTTATAAATCC-AAAACGGAAGCTACCTAA-CTGAATAGGTGTCAATGTGTTCATTGAACTGACAGAGTTAAACTCACGTGTGGA
Owl438b  40  ACAGAGAAATATCTGCTTATAAAACC-AAAACGGA-GCTTCCTAA-CTGCATGGTTGTCAATGTGTGCATTGAACTGACAGAGTTAAACTCACGTGTGGA

                 .........+.........+.........+.........+.........+.........+.........+.........+.........+.........2
             Con TTCAGCGGTTTGCAAACCCTTTCTGTGCAGAATCCCAAAACCGG-CATTTCCAGCCCAATGCCGGCATACGTACGAAAC-AGCAGTAA-ACTT-CGTCTA
             01  TTCAGCGGTTTGAAAACCCTTTCTGTGCAGAATCACAAAACCGG-CATTTCCAGCCCAATGCCGGCATACGTACGAAAC-AGCAGTGA-ACTC-CGTGTA
             02  TTTAGCAGTCTGCAAAACCTTTCTGTGCAGAATTCCAAAACTGG-CATTTCCAGCCCAATACCGGCATATGTACGAAAC-AGCGGTAAGACTT-CGTGTA
             03  TTCATCGGTTGGCAAACCCTTTCTGTGCAGAATCCCAAAACCGG-TATTTCCAGCCCAATGCAGGCATACTTACTAAAC-AGCAGGAA-ACTT-CGTCTA
             04  TTGAGCGTTGTGCAAACCCTTTCTGTGCAGAATCCCAAAACCGG-CATTTCCACCCCAATCCCGGCATACATACAAAAC-AGAGGTTAGACTT-CGTCTA
             05  TTCAGCGGTGTGCAAACAGTTTCTGTTCAGAATCCCGAAACCGG-CATTTCCAGCCCAATGCCTGGATACGTACGAAAC-AGCGGTAAGACTT-CGTCTA
             06  TTGAGCGTTGTGCAAACCCTTTCTGTGCAGAATCCCAAAACCGG-CATTTCCACCCCAATCCCGGCATACATACAAAAC-AGAGGTTAGACTT-CGTCTA
             07  TTCAGCGGTTTGCAAACCCTTTCTGTGCAGAATCCCCAAACCGG-CATTTCCAGCCCAATGCCGGCAGACGTACGAGAC-AGCGGTGA-ACTC-CGTGTA
             08  TTCAGCTGTTTGCAAACCCTTTCTGTGCAGAATCCTAAAACCGG-CATTTCCAGCCCAATGCCGGCATACGTACGAAAC-ACCGGTGA-ACTT-CGTGTA
             09  TTCAGCGGTTTCCAA-CCCCTTCCGGGCCGGATTCCCAAACCGG-TGTTTCCAGCCCAATGCCGGCATACATACTAAAC-AGCAGGAA-ACTT-CGTCTA
             10  TTCAGCGGCT-GCAAACCCTTTCTATGCAGAATCCCAAAACCGG-TAATTCCAGCCCAATACCGGCATACGTTCTAAAC-AGCAGGAA-ACTT-CGTCTA
             11  TTCAGCAGTTTGCAAACCCTTTCTGTGCAGAATCCCAAAACTGG-CATTTCCAGCCCAATATCGTCATACGTACGAAAC-AGCAGTAAGTCTT-CGGCTA
             12  TTCAGCAGGTTGCAAACCCTTTCTGTGCAGAATCCCAAAACAGG-CATTTTGAGGCCAATATCGTCATATGTACGATAC-AGCAGTAAGACTT-CGGCTA
             13  TTCAGCGGTGTGCAAATCCTTTCTGTGCAGAATCCGAAAACCGG-AATTTCCAGCCCCATGCCGGCATATGTACTAAAC-AGCAGTAA-ACTT-CGTCTA
             14  TTCAGCGGTTTGCAAATCCTTTCTGTGCAGAATCCCAAAACCGG-CATTTCCAGCCAAATGCCGGCATATGTACGAAAC-AGCAGTAA-ACTT-CGTCTA
             15  TTCAGCGGTTTGCAAACCCTTTCTGTGCAGAATCCCAAAACCGG-TATTTCCAGCCCAATGCCGGCATACGACCTAAAC-CGCAGGAA-ACTT-CGTCTA
             16  TTCAGCGGTTTGCAAACCCTTTCTGTGCAGAATCCCAAAACTGG-CTTTTCCAGCCCAATGCCGGCATACGTACGAAAC-AGCAGTGA-ACTT-CGTGTA
             17  TTCAGCGGTTTGCAAACCGTTTCTGTGCAGAATCCCAAATCCGG-TACTTCCAGCTCAATGCTGGCATATGTACTAAAC-AGCAGGAA-ACTT-CGTCTA
             18  TTCAGCGGTTTGTAAACCCTTTCTGTGCAGAATCCGAAAACCGG-AATTTCCAGCCCAATGCCGGCATACGTACGAAAC-AGCAGTAA-ACTT-CGTCTA
             19  TTCAGCGGTTTGTAAACCCTTTCTGTGCAGAATCCCAAAACCGG-AATTTCCAGCCCGATGCCGGCATACGTACGAAGC-AGCAGTAA-ACTT-CGTCTA
             20  TTCAGCGGTTTGCAAACCCTTTCTGTGCAGAATCCCAAAACCGG-CATTTCCAGCCCAATGCCGGCATACGTACGAAAC-AGCAGTGA-ACTT-CGTGTA
             21  TTCAGCGGTTTGCAAACCCTTTCTGTGCAGAATCCCAAAACCGG-CATTTCCAGCCCAATGCCGGCATACGTACGAAAC-AGCAGTGA-ACTT-CGTGTA
             22  TTCAGCGGTTTGCAAACCCTTTCTGTGCAGCATCCCAAAACCGG-CATTTCCAGCCCAATGCCGGCATACATACGAAAC-AGCAGTGA-ACTC-CGTGTA
             23  TTCAGCGGTTTGCAAACCCTTTCTGTGCAGAATCCCAGAACCGG-CATTTCCAGCCCAATGCCGGCATACGTACGAAAC-AGCAGTGA-ACTC-CGTGTA
             24  TTCAGCGGTTTCCAAACCCTTTCTGTGCAGAATCCCAAAACCGG-CATTTCCAGCCCAATGCCGGCATACGTACGAAAC-AGTAGTGA-ACTC-CGTGTA
             25  TTCAGCGGTTTGCAAACCCTTTCTGTGCAGAATCCCAAAACAGG-CATTTCCAGCCCAATGCCGGCATACGTACGAAAC-AGCAGTGA-ACTC-CGTGTA
             26  TTCAGCGGTTTGCAAACCCTTTCTGTGCAGAATCCCAAAACCGG-CATTTCCAGCCCAATGCCGGCATACGTACGAAAC-AGCGGTGA-ACTT-CGTGTA
             27  TTCAGCGGTTTGCAAACCCTTTCTGTGCAGAATCCCAAAACCGG-CATTTCCAGCCCAATGCCGGCATACGTACGAAAC-AGCAGTGA-ACTT-CGTGTA
             28  TTCAGCGGCT-GCAAACCCGTTCTATACAGAATCCCAAAACCGG-TATTTCCGGCCCAATACCGGCATACGTACTAAAC-AGCAGGAA-ACTT-CGTCTA
             29  TTCAGCGGTTTTCAAA-CCTTTCTGTGCAGAATCCCAAAACCGG-TAATTCCAGCCCAATGCCGGCATACGTACTAAAC-AGCAGTAA-ACTT-CGTATA
             30  TTCAGCGATTTGCAAACCCTTTCTGTGCAGAATCCCAAAACCGG-CATTTCCAGCCAAATGCCGGCATACGTACGAAAC-AGCAGTGA-ACTT-CGTGTA
             31  TTCAGCGGTTTGCAAACCCTTTCTGTGCAGAATCCCCATACCGGGCATTTCCAGCCCAATGCCGGCATATGTACGAAACCAGCAGTGA-ACTT-CGTGTA
             32  TTTAGCGGTGTGCAAACCCTTTCTGTGAAGAATCCCGAAACCGG-CATTTCCAGCCCAATGCCCTCATACGTACGAAAC-AGTGGTAAGACTT-CGTCTA
             33  TTCAGCGGTGTGCAACCC-TTTCTGTGCAGAATCCCAAAACCGG-CATTTCCTGACCAATGCCGGCCTACGTACGAAAC-AGCGGTAAGACTTTCATCTA
             34  TTCAGCGGTTTGCAAACCCATTCTGTGCAGAATCCCAAATCCGG-TATTTCCAGCCCAATGCCGGCATACGTACTAAAC-AGCAGGAA-ACTT-CGTCTA
             35  TTCAGCGGTTTGCAAACCCTTTCTGTGCAGAATCCCAAATCCGG-TATTTCCAGCCCAATGCCGGCATACGTACTAAAC-AGCAGGAA-ACTT-CGTCTA
             36  TCCAGCGGTTTGCAAACCCTTTCTGTGCATAATCCCTAA-CCGG-TATTTCCAGCCCAATGCCGGCATACGTACTAAAC-AGCAGGAA-ACTT-CTTCTA
             37  TTCAGCGGTTTGCAAACCCTTTCTGTGCACAAACCCAAAACCTG-TATTTCCAGCCCAATGCCGGCATACGTACTAAAC-AGCAGGAA-ACTT-TGTCTA
             38  TTCAGCGGTTTGCAAACCCTTTCTGTGCGTAATCCCAAAACCGG-CATT-CCTGCCCAATGCCCGCATACGTACGAAAA-ATCATTGA-ACTT-CTTGTA
             39  TTCAGCGGGTTGCAAACCCTTTCTGTGCAGAATCCCAAAACCGG-CATTTCCAGCCCAATGCCGGCATACGTACGAAAC-AGCGGTGA-ACTT-CGTGTA
             40  TTCAGCGGTTTGCAAACCCTTTCTGTGCAGAATCCCAAAACCGG-CATTTCCAGCCCAATGCCGGCATACGTACGAAAC-AGCAGTAA-ACTT-AGTCTG
```

Figure S1, page 5 of 12

Owl (n=40), 2 of 2

```
    .........+.........+.........+.........+.........+.........+.........+.........+.........+.........3
Con AAACTCAGGAGAAACGTTTCC-TTGAAACTGCTCTGCTGTGCGTT-CGTTCACCTAAGAGAGTTGAATCT-ATGTTGAGATTCAGCG-AGT-CAGAAACA
01  AATCTCAGGAGAAACGTTTCC-TTGAAACTGCTCTGCTGTGCGTC-CTTTCACCTAAGAGAGTTGAATCT-ATGTTGAGATTCAGCG-AGT-CAGAAACA
02  AAACTCAGGAGAAACGTTTCC-TTGTAACTGATCGTCTGTGCGTT-CGTTCAAGTAAGAGTGTTGAATCA-ATGTTTAGATTCAGCG-AGT-TAGAAACA
03  AAACTCAGGAGAAACGTTTCC-ATGAAACTGCTCTGCTGTGCGTT-CGTTCACCTAAGAGAGTTGAATCT-GTGTTGAGATTCAGCG-AGT-CAGAAACA
04  CAACTCAGGAGAAACGTTTCC-GTGAAACTGCTCTGCTGCGCGTT-CGTTCACCTAAGACAGTTGAATCT-ATGTTGAGATTCAGTG-AGT-TAGAAACA
05  AAACTCAGGAGAAACGTTTCC-GTGGAACTGCTCTGCCGTGCGTT-CGTTCACCTAAGAGAGTTGAATCT-ATGTTGAGATTCAGAG-AGT-TAGAAACA
06  CAACTCAGGAGAAACGTTTCC-GTGAAACTGCTCTGCTGCGCGTT-CGTTCACCTAAGACAGTTGAATCT-ATGTTGAGATTCAGTG-AGT-TAGAAACA
07  AATCTCAGGAGAAACGTTGCC-TTGAAACTGCTCTGCTGTGCGTT-CGTTCACCTAAGAGAGTTGAATCT-ATGTTGAGATCCAGCG-AGT-CCGGAACA
08  AATCTCAGGAGAAACGTTTCC-TTGAAACTGCTCTGCTCTGCGTT-CGTTCACCTCAGAGAGTTGAATCT-ATGTTGAGATTCAGCG-AGT-CAGAAACA
09  AAACTCAGGAGAAACGTTTCC-TTGAAACTGCTCTGCTGTGCGTT-CGTTCACCTAAGAGAGTTGAATCT-ATGTTGAGATTCAGCG-AGT-CAGAAACA
10  AAACTCAGGAGAAACATTTCC-TTGAAACTGTTCTGCTGTGCGTT-CGCTCACCTAAGAGAGTTGAATCT-ATGTTGAGATTCAGCG-AGT-CAGAACCA
11  AAACTCAGGAGAAACGTTTCC-TTGAAACGGATGTGCTGTGCGTT-CGTTCACCTAAGAGAGTTGAATCT-ATGTTTAGACTCAGCG-AGT-TAGAAACA
12  AAACTCAGGAGAAAAGTTTCG-TTGAAACTGATTTGCTGTGCGTT-CGTTCACCTAAGAGAGTTGAATAA-ATGTTTATTAACAGCG-AGT-TAGAAACA
13  AAACTCAGGAGAAACCATTCC-TTGCAACCGCTCTGCTGTGCGTT-CGTTCACCTAAGAGAGTTGAATCT-ATGTTGAGATTCAGCG-AGT-CAGAAACA
14  AACCTCAGGAGAAACGTTTCC-TTGAAACTGCTCTGCTGTGCCTT-CGTTCACATGAGAGAGTTGAATCT-ATGTTGAGATTCAGCG-AGT-CAGAAACT
15  AAACTCAGGAGGAACGTTTCC-TTGAAACTGCTCTGCTGTGCGTT-CGTTCACCTAAGAGAGTTGAATCT-ATGTTGAGATTCAGCG-AGT-CAGAAACA
16  AATCTCAGGAGAAACGTTTCC-TTGAAACTGCTCTGCTGTGCGTT-CGTTCACCTAAGAGAGTTGAATCT-ATGATGAGATTCAGCG-AGT-CAGAAACA
17  AAACTCAGGAGAAACGTTTCC-TTGAAACTGCTCTGCTGTGCGTT-CGTTCACCTAAGAGAGTTGAATCT-ATGTTGAGATTCAGCG-AGT-CAGAAACA
18  AAACTCAGGAGAAACGTTTCC-TTGAAACTGCTCTGCTGTGCATT-CGTTCACCGAAGAGAGTTGAATCT-ATGTTGAGATTCAGCG-AGT-CAGAAACA
19  AAACTCAGGAGAAACGTTTCC-TTGAAACCTCTCTGCTGTGCGTT-CGTTCACCTAAGAGAGTTGAATCT-ATGTTGAGATTCAGCG-AGT-CAGAA-CA
20  AATCTCAGGAGAAACGTTTCC-TTGAAACTGCTCTGCTGTGCGTT-CGTTCACCTAAGAGAGTTGAATCT-ATGTTGAGATTCAGCG-AGT-CAGAAACA
21  AATCTCAGGAGAAACGTTTCC-TTGAAACTGCTCTGCTGTGCGTC-CGTTCACCTAAGAGAGTTGAATCT-ATGTTGAGATTCAGCG-AGT-CAGAAACA
22  AATCTCAGGAGAAACGTTTCC-TTTAAACTGCTCTGCTGCGCGTT-CGTTCACCTAAGAGAGTTGAATCT-ATGTTGAGATTCAGCG-AGT-CAGAAACA
23  AATCTCAGGAGAAACGTTTCC-TTGAAAGTGCTCTGCTGTGCGTC-AGTTCACCTAAGAGAGTTGAATCT-ATGTTGAGATTCAGCG-AGT-CAGAAACA
24  AATCTCAGGAGAAACGTTTCC-TTGAAACTGCTCTGCTGTACGTT-CGTTCACCTAAGAGAGTTGAATCT-ATGTTGAGATTCAGCG-AGT-CAGAAACA
25  AATCTCAGGAGAAACGTTTCC-TTGAAACTGCTCTGCTGTGCGTT-CGTTCACCTAAGAGAGTTGAATCT-ACGTTGAGATTCAACG-AAT-CAGAAACA
26  AATCTCAGAAGAAACGTTTCC-TTGCAACTGCTCTGCTGTTCGTT-CGTTCACCTAAGAGAGTTGAATCT-ATGTTGGGATTCTGCG-AGT-CAGAAACA
27  AATCTCAGGAGAAACGTTTCC-TTGAAACTGCTCTGCTGTGCGTT-CGTTCACCTAAGAGAGTTGAATCT-ATGTTGAGATTCAGCG-AGT-CAGAAACA
28  AAACTCAGGAGAAACATTTCC-TTGAAACTGCTCTGCTGTGCGTT-CGTTCACCTAAGAGAGTTGAATCT-ATGTTGAGATTCAGCG-AGT-CAGAAACA
29  AAACTCAGGAGAAACGTTTCG-TTGAAACTGCTCTGCTGTGAGTT-CGTTCACCTAAGAGAGATGAATCT-ATGTAGAGGTTCAGCG-AGT-CAGAAACA
30  AATCTCAGGAGAAACGTTTCC-TTGAAACTGCTCTGCTGTGCGTT-CGTTCACCTAAGAGAGTTGAATCT-ATGTTCAGATTCAGCG-AGT-CAGAAACA
31  AATCTCAG-AGAAACGTTTCC-TTGAAACTGCTCTGCTGTGCGTT-CGTTCACCTAAGAGAGTTGAATCTTATGTTGAGATTCAGCGGAGT-CAGAAACA
32  AATCTCAGGAGATACATTTCC-GTGAAACTGCTCTGCTGTGCGTT-CCTCCCCCTAAGACAGTTGTATCT-ATGCTGAGACTCACTG-AGT-TAGAAACA
33  AAACTCCGGCGAAACGTTTCCCGTGAAACTGCTCTACTGTGCGTT-CGCTCACCTAAGAGAGTTGAATCG-ATTTTGAGATTCAGCG-AGT-TAGAAACA
34  AAACTCAGGAGAAACGTTTCC-TTGAAACTGCTCTGCAGTGCGTT-CCTTCACCTAAGAGAGTTGAATCT-ATGTTGAGATTCAGTG-AGT-CAGAAACA
35  AAACGCAGGAGAAACGTTTCC-TTGAAACTGCTCTGCTGTGCGTTTCCTTCACCTAAGAGAGTTGAATCT-ATGTTGAGATTCAGCG-AGTTCAGAAACA
36  AAACTCAGGAGGAACGTTTCC-TTGAAACTGCTCTGCTGTGCGTT-CGTTCACCTAAGAGAGTTGAATCT-ATGTTGAGATTCAGCG-AGT-CAGAAACA
37  AAACTCAGGAGGAACGTTTCC-TTGAATCTGCTCTGCTGTGCGTT-CGTTCACCTAAGAGAGTTGAATCT-ATGTTGAGATTCAGCG-AGT-CAGAAACA
38  AATCTCTGGAGTAACGTTTCC-TTTTCACTCCTCTGCTGAGCCAC-CGTTTCCCCAAGAGAAATCAATCT-AAGCTAAGATTCAGCG-AGT-CATAAACA
39  AATCTCAGGAGAAACGTTTCC-TTGAAACTGCTCTGCTGTGCGTT-CGTTCACCTAAGAGAGTTGAATCT-ATGTTGAGATTCAGCG-AGT-CAGAAACA
40  AAACTCAGGAGAAACGTTTCC-TTGAAACTGCTCTGCTGTGCGTT-CGATCACCTAAGAGAGTTGAATCT-ATGTTGAGATTCAGCG-AGT-CAGAAACA

    .........+.........+.........+.........+.........+.........+.........+.........+.........+.........4
Con C-GTTT--CTTCTAGG-AGCTG-CGTTGGG-ATATTCCC--GAGT-GAATGGGAC-CAT-AGGCT-GT
01  C-GTTT--CTTCTAGG-AGCTG-CGTTGGG-ATATTCCC--GAGT-GAATGGGAC-CAT-AGGCT-GT
02  C-GTTTC--TTCTAGG-AGCCG-CGTTGGG-ATATTCCC--GAGT-GAATGGGAC-CAT-AGGCT-GT
03  C-GTTTC--TTCTATG-AGCTG-CGTT-GGGATATTCCC--GAGT-GAATGGGAC-CAT-AGGCT-GT
04  C-GTTT--CTTCTAGG-AGCTG-CGTTGGG-ATATTCCC--GAGT-GAATGGGAC-CAT-AGGTT-GT
05  C-GTTT--CTTCTAGG-AGCTG-CGTTGGG-ATATTCCA--GAGA-GAACGGGAC-CAT-AGGCT-GT
06  C-GTTT--CTTCTAGG-AGCTG-CGTTGGG-ATATTCCC--GAGT-GAATGGGAC-CAT-AGGTT-GT
07  C-GTTT--CTTCTAGG-AGCTG-CGTTGGG-ATATTCCC--GAGT-GAATGGGA-CCAT-AGGCT-GT
08  C-GTTT--TTTCTAGG-AGCTG-CGTTGGG-ATATTCCC--GAGT-GAATGGGAC-CAT-AGGCT-GT
09  C-GTTTC--TTCTAGG-AGCTG-CGTT-GGGATATTCCC--GAGT-GAATGGGAC-CAT-AGGCT-GT
10  C-GTTTC--TTCTAGA-AGCTG-CGTT-GGGATATTCCC--GAGT-GAATGGGAC-CAT-AGGCT-GT
11  C-GTTTC--ATTTAGG-AGCTG-CATTGGG-ATATTCCC--GAGT-GAATGGGTC-CAT-ATGCT-GT
12  C-GTTTC--TTCTAGG-AACAG-TGTTGGG-ATATTTAC--GAGT-GAATGGGAC-CAC-AGACT-GT
13  C-GTTTC--TTCTGGG-AACTG-CGTT-GGGATATTCCC--GAGT-GAATGGGAC-CAT-AGGTT-GT
14  C-GTTT--CTTCCAGG-AGCTG-CGTTGGG-ATATTCCC--GAGT-GAATGGGAC-CAT-AGGCT-GT
15  C-GTTTC--TTCTAGG-AGCTG-CGTT-GGGATATTCCC--GAGT-GAATGGGAC-CAT-AGGCT-GT
16  C-GTTT--CTTCTAGG-AGCTG-CGTTGGG-ATATTCCC--GAGT-GAATGGGAC-CAT-AGGCT-GT
17  C-GATTC--TTCTAGG-AGCTG-CGTT-GGGATATTCCC--GAGA-GAATGGGAC-CAT-AGGCT-GT
18  C-GTTGA--TTCTAGG-AGCTG-CGTT-TCGATATTCCC--GAGT-GAACCGGAC-CAT-AGGCT-GT
19  C-GTTGC--TTCTAGG-AACTG-CGTT-GGGATATTCCC--GAGT-GAATGGGAC-CAT-AG-CT-GT
20  C-GTTT--CTTCTAGG-AGCTG-CGTTGGG-ATATTCCC--GAGT-GAATGGGAC-CAT-AGGCT-GT
21  C-ATTTTCTTCTTAGG-AGCTG-CGTTGGGTATATTCCC--GAGT-GAATGGGACTCAT-GGGCT-GT
22  C-GTTT--CTTCTAGG-AGCTG-CGTTGGG-ATATTCCC--GAGT-GAATGTGAC-CAT-AGGCT-GT
23  C-GTTT--CTTCTAGG-AGCTG-CGTTGGG-ATATTCCC--GAGT-GAATGGGAC-CAT-AGGCT-GT
24  C-GTTT--CTTCTAGG-AGCTG-CGTTGGT-ATATTCCC--GAGT-GAATGGGAC-CAT-AGGCT-GT
25  C-GTT---CTTCTAGG-AGCTG-CGTTGGG-ATATTCCC--GAGT-GAATGGGAC-CAT-AGGCT-GT
26  C-GTTT--CTCCTAGG-AGCTG-CGTTGGG-ATATTCCC--GAGT-GAATGGGAC-CAT-AGGCT-GT
27  C-GTTT--CTTCTAGG-AGCTC-CGTTGGG-ATATTCCC--GAGT-GAATGGGAC-CAT-AGGCT-GT
28  C-GTTTC--TTCTAGG-AGATG-CGTT-GGGATATTCCC--GAGT-GAATGGGAC-CAT-AGGCT-GT
29  C-GTTTC--TTCTGGG-GGCTG-CGTT-GGGATATTCCC--GAGT-GAATGGGAC-AAT-AGGCT-GT
30  C-GTTT--CTTCTAGG-AGCTG-CGTTGGG-ATATTACC--GAGT-GAATGGGAC-CAT-AGGCT-GT
31  CCGATT--CTTCTAGGGAGCTGGCGTTGGG-ATATTTCCCGAAGT-GAATGGGAACCATTAGGCT-GT
32  C-GTTT--CTTGTAGG-AGCTG-CGTTGGG-ATATTCCC--GAGT-GAATGGGAC-CAT-AGGCT-GT
33  C-GTTTT-CTTCTAGG-AGCAG-CGTTGGGGATATTCCC--GAGG-GAATATGAC-CAT-AGCTTTGT
34  C-GTTTC--TTCTAGG-AGCTG-CGTT-GGGATATTCCC--GAGT-GAATGGGAC-CAT-AGGCT-GT
35  C-GTTTC--TTCTAGG-AGCTG-CGTT-GGGATATTCCC--GAGTTGAATGGGAC-CAT-AGGCT-GT
36  C-GTTTC--TTCTAGG-AGCTG-CGTT-GGGATATTCCC--GAGT-GAATGGGAC-CAT-TCGCT-GT
37  C-GTTTTCTTTCTAGG-AGCTG-CGTTTGGGATATTCCC--GAGT-GAATGGGAC-CAT-AGGCT-GT
38  C-GTTT--CTTCTAGG-AGCTG-CGTTGGG-ATATTCCC--GAAT-GAATGGGAC-CAT-AGGCT-GG
39  C-GTTT--CTTCTAGG-AGCTG-CGTTGGG-ATATTCCC--GAGT-GAATGGGAC-CAT-AGGCT-GT
40  C-GTTT--CTTCTAGG-AGCTG-CGTTGGG-ATATTCCC--GAGA-GAATGGGAC-CAT-AGGCT-GT
```

Figure S1, page 6 of 12

Spi (n=41), 1 of 2

```
               .........+.........+.........+.........+.........+.........+.........+.........+.........+.........1
          Con --ACAGAGGAAT-ATCT-CGCTCTAAAA-CCAAAACGG-AGCAA-TCTAACTGAATG-CTTGTC-AATGTGTGCATT-CAA-CTTAC-AGAGTTAAACT-
Spi01a    01  --ATAGAGAAAT-ATCT-CCCTCTAAAA-CCAAAACGGGAGCAA-TCTAACTGAATG-CTTGTC-AATGGGTGCATT-CAT-CTTAC-AGAGTTAAACT-
Spi01b    02  --ACAGAGAAAT-ATCT-CGCTCTAAAA-CCAAAACGG-AGCAT-TCTAACTGAATG-CTTGTC-AATGTGTGCATT-CAT-CTTAC-AGAGTTAAACT-
Spi02a    03  --ACAGAGGAAT-ATCT-CGCTCTAAAA-CCAAAACGG-AGCAA-TCTACCTGAATG-CTTGTC-AATGTGTGCATT-CAC-CTTAC-AGAGTTACACT-
Spi03a    04  ATCCAGAGGAAT-ATCT-CGCTCTTAAACACCAAACGA--GACA-TCTACTGGAATG-CTGGTCAAGTGTGGGCATT-CCA-ATTGC-AGAGATAAACTG
Spi03b    05  --ACAGAGGAAT-ATCT-CGCTCTAAAA-CCAAAACGGGAGCAA-TCTAACTGAATG-CTTGTCCAATGTGTGCATTTCAATTTAC-AGAGTTTAAAA-
Spi05a    06  --CAGGAGGAAT-ATCT--GCTCTAAAA-CCCAAACGG-AGCAA-TCTA-CTGAATGGCTTGTC-AATGTGTGCCTTTCAA-CTTACCAGAGTTAAACT-
Spi05b    07  --ACAGAGGAAT-ATCT-CGCTCTAAAA-CCAAAACGG-AGCAA-TCTAACTGAATG-CTTGTC-AATGTGTGCATT-CAA-CTTAC-AGAGTTACACT-
Spi06a    08  --ACAGAGGAAT-ATCT-CGCTCTAAAA-CCAAAACGG-AGCAA-TCTAACTGAATG-CTTGTC-AATGTGTGCGTT-CAA-CTTAC-AGAGCTAAACT-
Spi07a    09  --AAAGAGAATTTATCTTCGCCCTAAAACCAAAA--CGGAGCAATT-TAAATGAAAG-CTTGTCAAT-GTGTACATT-CAA-CTTAG-AGAGTTAAA-CT
Spi08a    10  -ACAGAGGAAAT-ATCT-CGCTCTAAAACCCAAAACGG-AGCAAATCTAACTGAATG-CTTGTC-AATGTGTGCATTCAAA-CTTAC-AGAGTTAAACT-
Spi09a    11  -ACAGAGGGAAT-ATCT-CGCTCTAAA--CCAAAACGG-AGCAA-TCTAACTGAATG-CT-GTC-AACGTGTGCATTCA---CTTAC-AGAGTTAAACT-
Spi10a    12  --ACAGAGGAAT-ATCT-CGCTCTAAAA-CCAAAACGG-AGCAA-TCTAACTGAATG-CTTGTC-AATGTGTGCATT-CAA-CTTAC-AGAGTTAAACT-
Spi10b    13  --ACAGAGGAAT-ATCT-CGCTCTAAAACCTAAACGGA--GCAA-TCTAACTAAATG-CTTGTCAAT-GTGTGCATT-CAA-CTTAC-AGAGATAAACT-
Spi12a    14  --ACAGAGGAAT-ATCT-CGCTCTAAAA-CCAAAACGG-AGCAA-ACTAACTGAATG-CTTGTC-AATGTGTGCATT-CAA-CTTAC-AGAGTTACACT-
Spi14a    15  --AAAGAGAAAT-ATCT-CGCTCTAAAAGCAAAA--CGGAGCAATC-TAACTGAAAG-CTTGTCAAT-GTGTACATT-CAA-CTTAC-ACAGTTCAA-CT
Spi15a    16  --ACAGAGGAAT-ATCT-CGCTCTAAAA-CGAAAACGG-AGCAA-TCTAACTGAATG-CTTGTC-AATGTATGCATT-AAA-CTTAC-AGAGTTAAACT-
Spi15b    17  --ACAGATTAAT-ATCT-CGCTCTAAAA-CCAAAGCGG-AGCAA-TCTAACTGAATG-CTTGTC-AATGTGTGCATT-CAA-CTTAC-AGAGTTAAACT-
Spi16a    18  --ACAGAGAAAT-ATTCTCGCTTTTAATTCGAAAC-GGAGGCAATCGTAACCGAAAGCTTGGTCAAA-GTGTACATT-CAA-CTTAC-AGAGTTAAAGCT
Spi16b    19  --AAAGAGAAAT-ATCT-CGCTCTGAAACCAAAA--CGGAGCAATA-TAACTGAAAG-CTTGTCAAT-GTGTACATT-CAA-GATAT-AGAGTTAAA-CT
Spi17a    20  --ACAGAGAAAT-ATCT-CGGTCTAAAA-CCAAAACGG-AGCAA-TCTAACTGAATG-CTTGTC-AATGTGTGCATTCAA--CTTAC-AGAGTTAAACT-
Spi19a    21  --ACAGAGGAAT-ATCT-AGCTCTAAAA-CCAAAACGG-AGCAA-TCTAACTGAATG-CTTGTC-AATGTGTGCATT-CAA-CTTAC-AGAGTTAAACT-
Spi21a    22  --ACAGAGGAAT-ATCT-CGCTCTAAAA-CCAAAACGG-AGCAA-TCTAACTGAATG-CTTGTC-AATGTGTGCATT-CAA-CTTAC-AGAGTTAAACT-
Spi22a    23  --AAAGAGAAAT-ATCT-CGCTCTAAAACCAAAA--CGGAGCAATC-TAACTGAAAG-GTTGTCAAT-GTGTACATT-CAA-CTTAC-AGAGTTAAA-CT
Spi22b    24  --AAAGAGAAAT-ATCT-CGCTCTAAAACCAAAA--CGGAGCTACC-TAACTGAAAG-CTTGACAAG-GCGTACATT-CAA-CTTAC-AGAGATAAA-CT
Spi23a    25  --AAAGAAAAAT-ATCT-CGCTCT-AAACCAAAA--CGGAGCA-TC-TAACTGAAAGGCTTGTCAAT-GTGTATATT-CAT-CTTAC-AGAGTTAAG--T
Spi23b    26  --AAAGAGAAAT-ATCT-CGCTCTAAAACCAAAA--CGGAGCAATC-TAACTGAAAG-CTTGACAAATGTGTACATT-CAA-CTTAC-AGAGTTAAA-CA
Spi24a    27  --ACAGAGGAAT-ATCT-CGCTCTAAAA-CCAAAATGG-AGCAA-TCTAACTGAATG-CTTGTC-AATGTGTGCCTT-CAA-CTTAC-AGAGTTAAACT-
Spi24b    28  --ACAGAGGAAT-ATCT-CGCTCTAAAA-CCAAAACGG-AGCAA-TCTAACTGAATG-CTTGTC-AATGTGTGCGTT-CAG-CTTAC-AGAGCTAAACT-
Spi25a    29  --ACAGAGGAAT-ATCT-CGCTCTAAAA-CCAAAACGG-AGCAA-TCTAAGTGAATG-CT-GTC-AATGTGTGCATTCAA--CTTAC-AGAGTTAAAGT-
Spi25b    30  --ACAGAGGAAT-ATCT-CGCTCTAAAA-CCAAAACGG-AGCAA-TCTAACTGAATG-CTTGTC-AATGTGTCCATTCAA--CTTAC-AGAGTTAAAGT-
Spi26a    31  --AAAGAGAAAT-ATCT-CGCTCTAAAACCAAAA--CGGAGCAATC-TAACTGAAAG-CTTGTCAAT-ATGTACATT-CAA-CTTAC-ACAGTTCAA-CT
Spi27a    32  --AAAGAGAAAT-ATCT-CGCTCTAAAACCAAAA--CGGAGCAATC-TAACTGAAAG-CTTGTCAAT-GTGTACATT-CAA-CTTAC-ACAGTTCAA-CT
Spi28a    33  --ACAGAGGAAT-ATCT-CGCTCTAAAA-CCAAAACGG-AGCAA-TCTAACTGAATG-CTTGTC-AATGTGTGCGTT-CAA-CTTAC-AGAGTTAAACT-
Spi32a    34  --ACAGAGGAAT-ATCT-CGCTCTAAAA-CCAAAACGG-AGCAA-TCTAACTGAATG-CTTGTC-AATGTGTGCATT-CAA-CTTAC-AGAGTTAAACT-
Spi33a    35  --ACAGAGGAAT-ATCT-CGCTCTAAAA-CCAAAACGG-AGCAA-TCTAACTGAATG-CTTGTC-AATGTGTGCATT-CAA-CTTAC-AGAGTTAAACT-
Spi34a    36  --AAAGAGAAAT-ATCT-CGCTCTAAAA-CCAAAACGG-AGCAA--ATTAAGGAAGG-CTTGTC-AATGTGTGCCTT-CAA-CTTAC--AGATTAGACT-
Spi37a    37  --AAAGAGAAAT-ATCT-CGCTCTAAAAAAAAAAAACGGAGCAATC-TAACTGAAAG-CTTGTCAAT-GTGTACATT-CGA-CTTAC-AGAGTTAAA-CT
Spi40a    38  --ACAGAGGAAT-ATCT-CGCTCTAAAA-CCGAAACGG-AGCAA-TCTAACTGAATG-CTTGTC-AATGTGTGCATT-CAT-CTTAC-AGAGTTAAACT-
Spi41a    39  -GACAGAGGAAT-ATCT-CGGTCT-AAATCCAAAACGA--GCAA-TCTACTG-AATT-CTTGTCAAA-GAGAGCATT-CAA-CTTAC-AGAGGTAAACT-
Spi41b    40  --ACAGAGGAAT-ATCT-CGCTCTAAAA-CCAAAACGG-AGCAA-TCTAACTGAATG-CTTGTC-AATGTGTGCATT-CAA-CTTAC-AGAGTTAAACT-
Spi42a    41  ---AAAAAAAAT-ATCT-CGCTCTAAAACCAAAA--CGGAGCAATC-TAACTGAAAC-CTTGTCAAT-GTGTACATT-CAA-CTTAC-AGAGTTACT-CT

              .........+.........+.........+.........+.........+.........+.........+.........+.........+.........2
          Con CGTG--TGTGTTTGCAGCAGTTT-AGAAACCC--TTTCTGTGCAGAGATGCAAAA-CTG-TCATTT-CCAGCC--AAATCCAGGCACATAAG-AAAAA-G
          01  CGTG--TGTGTTTGCTGCAGTTT-AGAAACCC--TTTCTGTGCAGAGATGCAAAA-CTG-TCATTT-CCAGCC--AAATCCAGGCACATAAG-GAAAAAG
          02  CGTG--TGTGTTTGCTGCAGTTT-AGAAACCC--TTTCTGTGCAGAGATGCAAAA-CTG-TCATTT-CCAGCC--AATTCCAGGCACATAAG-GAGAA-G
          03  CGTG--TGTGTTTGCAGCAGTTT-AGAAACCC--TTTCTCTGCAGAGATGCAAAA-GTG-TCATTT-CCAGCC--AAATCCAGGCACATAAG-AAAAA-G
          04  CGTG--TGT-TTGGCAGCAGTTT-AGAAACCC--TTTCTGTGCAGAAATGCAAAA-CTGTTCACTT-GCAGCCCAAATCCCAGGCACATAAG-GAAAA-G
          05  TGGGTGTGTGTTTACAGCAGTTT-AGAAAACCCTTTCTGTGCAGAGATGCAAAA-CTG-TCATTT-CCAGCC--AAATCCAGGCACATAAG-GAAAA-G
          06  GGGGG-TGTGTT-GCAGCAGTTT-AGAAACCC--TTTCTGTGCAGAGATGCAAAAAGTG-TCATTT-CCAGCCC-AAATCCAGGCACATTAG-AAAAA-G
          07  CGTG--TGTGTTTGCAGCAGTTT-AGAAACCC--TTTCTGTGCAGAGATGCAAAA-GTG-TCATTT-CCAGAC--AAATCCAGGCACATAAG-AAAAA-G
          08  CGTG--TGTGTTTGCAGCAGTTT-AGAAACCC--TTTCTGCGCAGAGATGCAAAA-GTG-TCATTT-CCAGGC--AAATCCAGGCACATAAG-AAAAA-G
          09  CGTG--CGTGTTTGCAGCAGTTTTAGAAACCC-TTT-CTGTGCAGAGATGCAAAA-GTA-TCATTT-CCAGAC--AAATCCAGGCACATAAG-AAAAA-G
          10  CGTG--TGTGTTTGCAGCAGTTT-AGAAACCC--TTTCTGTGCAGAGATGCAAAA-GTG-TCATTT-CCAGCC--AAATCCAGGCACGTAAG-AAAAA-G
          11  CGTG--TGTGTT-GCAGCAGTTT-AGAAACCC--TTTCTGTGCAGAGATGCAAAA-GTG-TCATTT-CCAGCC--AAATCCAGGCACATAAG-AAAAAAG
          12  CGTG--TGTGTTTGCAGCAGTTT-AGCAACCC--TTTCTGTGCAAAAATGCAAAA-CTG-TCATTT-CCAGCC--AAAACCAGGCACAAACT-GTAAAAG
          13  CATG--TGTGTTTGCAGAAGTTT-AGAAACTC--TTTCTGTGCGGAGACGCAAAA-CTGTC-ATTT-CCAGCCC--AATCCGGGCACATCAG-AAAAA-G
          14  GGTG--TGTGTTTGCAGCAATTT-AAAAACCC--TTTCTATGCATAGATGCAATA-GTG-TCATTT-CCAGCC--AACTCCAGGCACATAAG-AAAAA-A
          15  AGTG--TGTGTTTGCAGCAGTTT-AGAAACCC-TTTTCTGTGCAGAGATGCAAAA-CTG-TCATTT-CCAGAC--AAATCCAGGCACATTAG-AAAAA-G
          16  CGTG--TGTGTTTGCAGCAGTTT-AGAAACCC--TTTCAGTGCAGAGATGCAAAA-CTG-TCATTT-CCAGCC--AAATCCAGGCACAT-CA-GAAAAAG
          17  CGTG--TGTGTTTGCAGCAGTTT-AGAAACCC--TTTCTGTGCAGAGATGCAAAA-CTG-TCATTT-CCAGCC--AAATCCAGGCACAT-CA-CAAAAAG
          18  CGTG--TGTGTT-GCAGCATGTTTAGAAACCC-TTTTTTG-GCAGAGATGCAAAA-CTG-TCATTTTCCAGAC--AAATTCAGGCACATAAG--AAAAAG
          19  CGTG--TGTGTTTGCAGCAGTTT-AGAATCCC-TTT-CTGTGCAGAGATGCAAAA-CTG-TCATTT-CCAGAC--AAATCCAGGCGCATAAG-AAAAAAG
          20  CGTGTGTGT-TTTGCAGCAGTTT-AGAAACCC--TTTCTGTGCAGAGATGCAAAA-CTG-TCATTT-CCAGCC--AAATCCTGGCACATAAG-GAAAA-G
          21  CGTG--TGTGTTTGCAGCAGTTT-AGAAACCC--TTTCTGTGCAGAGATGCAAAA-CTG-TCATTT-CCAGCC--AAATCCAGGCACGT-CA-GAAAAAG
          22  CGTG--TGTGTTTGCAGCAGTTT-AGAAACCC--TTTCTGTGCAGAGATGCAAAA-GTGGTCATTT-CCAGCC--AATTCCAGGCACATAAG-AAAAA-G
          23  CGTG--TGTGTTTGCAGCAGTTT-AGAAACCC-TTT-CTGTGCAGAGATGAAAAA-CTG-TCATTT-CCAGAC--AAATCCAGGCACATAAG--AAAAAG
          24  CGTG--TGTGTTTGCAGCAGTTT-AGAAACCC-TTT-CTGTGCAGTGATGCAAAA-CTG-TCATTT-CCAGTC--AAATCCAGGCACATAAG---AAAAG
          25  CGTG--TGTGTTTGCAGCAGTTTAGAAAACCC-TTT-CTGTGCAGAGTTGCAAAA-CTG-TCATTT-CCAGAG--AAATCCAGGCACATAAG-AAAAA-G
          26  CGTG--TGTGTTTGCAGCACTTT-AGAAACCC-TTT-CTGTGCAGAGATGCAAAA-CTG-TCATTT-CCAGAC--ATATCCAGGCACATAAGTAAAAAAG
          27  CGGG--TGTGTTTGCAGCAGTTT-AGAAACCC--TTTCTGTGCAGAGATGCAAAA-GTG-TCATTT-CCAGCC--AAATCCAGGCACATAAG-AAAAA-G
          28  CGTG--TGTGTTTGCAGCAGTTT-AGAAACCC--TTTCTGTGCAGAGATGCAAAA-GTG-TCATTT-CCAGCC--AAATCCAGGCACATAAG-AAAAA-G
          29  CG--TGTGTGTTTGCAGCAGTTT-AGAAACCC--TTTCTGTGCAGAGATGCAAAA-CTG-TCATTT-CCAGCC--AAATCCAGGCACATAAG-GAAAA-G
          30  CG--TGTGTGTTTGCAGCACTTT-AGAAATCC--TTTCTGTGCAGAGATGCAAAA-CTG-TCATTT-CCAGCC--AAATCCAGGCACATAAG-GAAAA-G
          31  GGTG--TGTGTTTGCAGCAGTTT-AGAAACCC-TTT-CTGTGCAGAGATGCAAAA-CTG-TCATTT-CCAGAC--AAATCCAGGCACATAAG-AAAAA-G
          32  AGTG--TGTGTTTGCAGCAGTTT-AGAAACCC-TTT-CTGTGCAGAGATGCAAAA-CTG-TCATTT-CCAGAC--AAATCCAGGCACATAAG-AAAAA-G
          33  CGTG--TGTGTTTGCAGCAGTTT-AGAAACCC--TTTCTGTGCAGAGATGCAAAA-GTG-TCATTT-CCAGCC--AAATCCAGGCACCTAAT-AAAAA-G
          34  CGTG--TGGGTTTGCAGCAGTTT-AGAAACCC--TTTCTGTGCAGAGATGCAAAA-CTG-TCATTT-CCAGCC--AAATCCAGGCACAT-CA-GAAAAAG
          35  CGTG--TGTGTTTGCAGCACTTT-AGAAACCC--TTTGTGTGCAAAGATGCAAAA-GTG-TCATTC-CCAACC--AAATAAAGGCACATAAG-AAAAA-G
          36  CGTG--TGTGTTTGCAGCAGTTT-AGAAACCC--TTTCTGTGCAGAGATGCAAAA-CTG-TCATTT-CCAGCC--AAATCCAGGCACATAAG-AAAAA-G
          37  TGTG--TTTGTTTGCAGCAGTTT-AGAAACAA-TTT-CTATGCACAGATGGAAAA-CTG-TCATTT-CCAGAC--AAATCCAGGCACATAAG-AAAAA-G
          38  CGTG--TGTGTTTGCAGCAGTTT-AGAAACCC--TTTCTGTGCAGAGATGCAAAA-GTG-TCATTT-CCAGCC--AAATCCAGGCACATAAG-AAAAA-G
          39  CGTG--TGT-ATTGCAGCAGTTT-AGAAGCCC--TTTCAGTGCGGAGATGCAAAA-CTATCAA--T-ACAAGC--AAATCCAGGCACATCAG-AAAAA-G
          40  CGTG--TGTGTTTGCAGCAGTTT-AGAAACCC--TTTCTGTGCAGAGATGCAAAA-CTG-TCATTC-CCAGCC--AAATCCAGGCACAT-CA-GAAAAAG
          41  CGTG--TGTGTTTTCAGCAGTTT-AGGAACCC-TTT-CTGTGCAGTAATGCAAAT-CTG-TCATTT-CCAGAC--AAATCCAGGAACATAAG-AAAAA-G
```

Figure S1, page 7 of 12

Spi (n=41), 2 of 2

```
    .........+.........+.........+.........+.........+.........+.........+.........+.........+.........3
Con CAGTAATAGT-GCGT--CTAAAACACAGAAAGAAACGCATCATT-CAAACTGCTCTGCTGTGTGTT-CGTTTCAACTAAGGG-AGCTGAAGCTGAGTTT-
01  CAGTATTAGT-GCGT--CTAAAACACAGA-AGAAACGCATCATT-CAAACTGCTCTGCTGTGTGTT-CGTTTCAGCTAAGGG-AGCTGAAGCTGAGTTT-
02  CAGTAATAGT-GCGT--CTAAAACACAGA-AGAAACGCATCATT-CAAACTGCTCTGCTGTGTGTT-CGTTTCAGCTGAGGG-AGCTGAAGCTGAGTTT-
03  CAGTAATAGT-GCTT--CTAAAACACAGA-AGAAACGCATCATT-CAAACTGCTCTGCTGTGTGTT-CGTTTCAACTAAGGG-AGCTGAAGCTGAGTTT-
04  CAGTAATAGT-GCAT--CTAAAACACAGAAGAAAACGCATCATT-CAAACTGCTCTCCTGTGTGTT-CGTTTCAGCTAAGGG-AGCTGAAGCTGAGTTT-
05  CAGTAATAGT-GCGT--CTAAAACACAGA-AGAATCGCATCATT-CAAACTGCTCTGCTGTGTGTT-CGTTTCAGCTAAGGG-AGCTGAAGCTGAGTTT-
06  CAGTAATAGT-GCGT-CTTAAAACACAGAAGAAAACGCATCATTCAAAACTTCTCTGCTGTGTGTT-CGTTTCAAATAAGGG-AGCTGAAGCTGAGTTT-
07  CAGTAATAGT-GCGT--CTAAAACACAGA-AGAAACGCATCATT-CAAAGTGCTCTGCTGTGTGTT-CGTTTCAACTAAGGG-AGCTGAAGCTGAGTTT-
08  CAGTAATAGT-GCGTTCTAAAAACACAGA-AGAAACGCATCGTTTCAAACTGCTCTGCTGTGTGTTTCGTTTCAACTAAGGG-AGCTGAAGCTGATTTT-
09  CAGAATTAGT-GCGT--CTAAAACACAGAA-GAAACGCATCATT-CAAACTGCTCTGCTGTGTGTT-CGTTTCAACTAAGGG-AGATGTAGCTAT-GTTT
10  CAGTAATAGT-GCGT-CT-AAAACACAGAAGAAA-CGCATCATTCAAA-CTGCTCTGCTGTGTGTT-CGTTTCAACTAAGGG-AGCTGAAGCTGAGTTT-
11  CAGTAATAGT-GCGT-CT-AAAACACAGAAGAAA-CGCATCATTCAAA-CTGCTCTGCTGTGTGTT-CGTTTCAACTAAGGG-AGCTGAAGCTGAGTTT-
12  CAGTAATAGT-GCGT--CTAAAACACAGA-AGAAACGCATCATT-CAAACTGCTCTGCTGTGTGTT-CGTTTCAACTAAGGG-AGCTGAAGCTGAGTTT-
13  TAGTAATAGT-GCGT--CTAAAATACTAGAACAAACGCATCATT-CAA-CTGCTCTGCTGTGTGTC-CGTTCCACCTAAGGGGAGCTGAAGCTGAGTTT-
14  CAGTAATAGT-GCGT--CTAAA-CACAAATAGAAACGCATCATT-CAAACTGCTCTGCTGTGTGTT-CGTTTCAACTAAGGG-AGCTGAAGCTGAGTTT-
15  CAGAATTAGTTGCGT--CTAAA-CACAGAA-GAAACGCATCATT-CAAACTGCTCTGCTGTGTGTT-CGTTTCAACTAAGGG-AGCTGTATCTGT-GTTT
16  CAGTAATAGT-GCGT--CCAAACCTCAGA-AGAAACGCATCATT-CAAACTACTCTGCTGTGTGTT-CGTTTCAACTAAGGG-AGATGAAGCTGAGTTT-
17  CAGTAATAGT-GCGT--CTAAA-CATAGA-AGAA-TGCATCATT-CAAACTGCTCTGCTGTGTGTT-CGTTTCAACTAAAGG-AGCTGAAGCTGAGTTT-
18  CAGAATTAGT-GCGT--CTAAAACAGAGAA-GAAACGCATCATT-CAAACTGCTCTGCTGTGTGTT-CGTTTCAACTAAGGG-AGCTGTAGCTGT-GTTT
19  CAGAATTAGT-GCGT--CTAAAACCCAGAA-GAAACGCATCAGA-CAAACTGCTCTGCTGTGTGTT-CGTTTCAACTAAGGG-AGCTGTAGCTGT-GTTT
20  CAGTAATAGT-GCGT--CTAAAACACAGA-AGAAACGCATCATT-CAAACTGCTCTGCTGTGTGTT-CGTTTCAGCTAAGGG-AGCTGAAGCTGCCTTT-
21  CAGTAATAGC-GCGT--CTAAAACACAGA-AGAAACGCATCATT-CAAACTGCTCTGCTGTGTGTT-CGTTTCAACTAAAGGGAGCTGAAGCGGAGTTTT
22  CAGGAATACT-GCGT--CTAAAACACAGA-AGACACGCATCATT-CAAACTGCTCTGCTGTGTGTT-CGTTTCAACTAAGGG-AGCTGAAGCTGAGTTT-
23  CAGAATTAGT-GCGT--CTAAAACACAGAA-GAAACGCATCATT-CAAACTGCTCTGCTGTGTGTT-CGTTTCAACTAAGGG-AGCTGAAGCTGT-GTTT
24  CAGAATTAGT-GCGT--CTAAAACACAGAA-GAAACGCATCATT-TCAACTGCTCTGCTGTGTGTT-CGTTTCAGCTAAAGG-AGCTGTAGCTGT-GTTT
25  CAGA-TTAGT-GCGT--GTAAAACACAGAA-GAAACGCATCATT-CAAACTGCTCTGCTGTGTGTT-CGTTTCAACTAAGGG-AGCTGTAGCTGT-GTTT
26  CAGAATTAGT-GCGT--CTAAAACACAGAA-GAAACGCATCATT-CAAACTGCTCTACTGTGTGTT-CGTTTCAACTAAGGG-AGCTGTAGCTGT-GTTT
27  CAGTATTAGT-GCGT-CT-AAAACACAGAAGAAA-CGCATCATTCAAA-CTGCTCTGCTGTGTGTT-CGTTTCAACTAAGGG-AGCTGAAGCTGAGTTT-
28  CAGTAATAGTTGCGT-CT-AAAACACAAAAGAAA-CGCATCATTCAAA-CTGCTCTGCTGTGTGTT-CGTTTCAACTAACGG-AGCTGA-GCTGAGTTT-
29  CAGTATTAGT-GCGT--CTAAAACACAGA-AGAAACGCATCATT-CAAACTGCTCTACTGTGTGTT-CGTTTCAGCTAAGGG-AGCTGAAGCTGAGTTT-
30  CAGTAATAGT-GCGT--CTAAAACACAGA-AGAAACGCATCATT-CAAACTGCTCTGCTGTGTGTT-CGTTTCAGCTAAGGG-AGCTGAAGCTGAGTTT-
31  CAGAATTAGT-GCGT--CTAAAACACAGAA-GAAACGCATCATT-CAAACTACTCTGCTGTGTGTT-CGTTTCAACTCAGGG-AGCTGTATCTGT-GTTT
32  CAGAATTAGT-GCGT--CTAAAACACAGAA-GAAACGCATCATT-CAAACTGCTCTGCTGTGTGTT-CGTTTCAACTAAGGG-AGCTGTAGCTGT-GTTT
33  CAGTAATAGT-GCGT-CT-AAAACACAGAAGAAA-CGCATCATTCAAA-CTGCTCTGCTGTGTGTT-CGTTTCAACTAAGGG-AGCTGAAGCTGAGTTT-
34  CAGTAATAGT-GCGT--CTAAAACACAGA-AGAAACGCATCATT-CAAACTGCTCTGCTGTGTGTT-CGTTTCAACTAAGGA-AGCTGAAGCTGAGTTT-
35  GAGTAATACT-GCGT-CTAAAAACACAGA-AGAAACGCATCATT-CAAACTACTCCGCTGTGTGTT-CGTTTCAACTAAGGG-AGCTGAACCTGAGTTT-
36  CAGTAATAGT-GTGT--CTAAAACACAGA-AGAAGTGCATCATT-CAAACTGCTCTGTTGTGTGTT-TGTTTCAACTAAGGG-AGCTGAAGCTGTGTTT-
37  CAGAATTAGT-GCGT--CCAACACACAAAAAGAAACGCATCATT-CAAACTGCTCTGCTGCGTGTT-CGTTTCAACTAAGGG-AGCTGTAACTTTTGTTT
38  CAGTAATAGT-GCGT-CT-AAAACACAGAAGAAA-CGCATCATTCAAA-CTGCTCTGCTGTGTGTT-CGTTTCAACTAAGGG-AGCTGAAGCTGAGTTT-
39  CAGTAATAGT-GCGT--CTAAACCTCAGAAGAAT-CACATCATT-CAAACTGCTCTGCTGTGTGTT-CGTTTCAGCTAAGGG-AGCTGAAGCTGAGTTT-
40  CAGTAATAGT-GCGT--CTAAAACACAGA-AGAAACGCATCATT-CAAACTGCTCTGCTGTGTGTT-CCTTTCAACTAAGGG-AGTTGAAGTAGAGTTT-
41  CAGAATTAGT-GCGT--CTAAAACACAGAA-GAAACGCATCATT-CAAACTGTTCTGCTGGGTGTT-CGTTTCAACTAAGGG-AGCTGTAGCTGT-GTTT

    .........+.........+.........+.........+.........+.........+.........+.........+.........+.........4
Con CA--TTCAGCGAGTT-AGCAA-CACGTTT--CTTCTA-GGAACTGCGTTT-GGATTTTCC-AGAGCGAAA-GGGAGCATTT-GCTGT-
01  CA--TTAAGCGAGAT-AGCAA-CACGTTT--CTTCTA-GGAACTGCGTTT-GGATTTTCC-AGAGTGAAA-CGGAGCAGAT-GCTGT-
02  CA--TTCAGCGAGTT-AGCAA-CACGTTT--CTTCTA-GGAACTGCATTT-GGATTTTCC-AGAGCGAAA-GGGAGCATAT-GCTGT-
03  CA--TTCAGCGAGTT-AGCAA-CACGTTT--CTTCTA-GGAACTGCGTTT-GGATTTT-CCAGAGCGAAA-GGCAGCATTT-GCTGT-
04  CA--TTCAGCGAGTT-AGCAAACACGTTTC-T-TCTAGGAACT-GCGTTT-GGATTTTCC-AGAGCGAAA-GGAAGCATTT-GCTGT-
05  CA--TTCAGTGAGTT-AGCAA-CACGTTT--CTTCTA-GGAACTGCGTTT-GGATTTTCC-AGAGCGAAA-GGCAGCATTT-GCTGT-
06  CA--TTCAGCGAGTT-AGCAA-CACGTTT--CTTCTA-GGAACTGCGTTT-GGATTTTCC-AGAGCGAAA-GGCAGCATTT-GCTGT-
07  CA--TTCAGCGAGTT-AGCAA-CACGTTT--CTTCTA-GGAACTGCGTTT-GAATTTT-CCAGAGCGAAA-GGCAGCATTT-GCTGT-
08  CA-TTTCAGCGAGTT-AGCAA-CACGTTTT-CTTCTAGGAAACTGCGTTT-GGATTTTTCCAGAGCGAAAAGGCAGCATTTTGCTGTT
09  CA-CT-CACCGAGTT-AGCAA-CACGTTT--CTTCTAGGAACTG-CGCTTGGA-TATTCC-CGTGCGAAA-GGGTGCGTA-TGCTGT-
10  CA--TTCAGCGAGTT-AGCAA-CACGTTT--CTTCTA-GGAACTGCGTTT-GGATTTTCC-AGAGCAAAA-GGCAGCATTT-GCTGT-
11  CA--TTCAGCGAGTT-AGCAA-CACGTTT--CTTCTA-GGAACTGCGTTT-GGATTTTCC-AGAGCGAAA-GGCAGCATTT-GCTCT-
12  CA--TTCAGCGAGTT-AGCAA-CACGTTT--CTTGTA-GGAACTGCCTTT-GGATTCTCC-AGAGCGAAA-GGGAGCATTT-GCTGT-
13  CT--TTCATCGAGTT-AGCAA-CACGTTTT-TCTCTTGGAACTTGCGTTT-GGAATTCC---GAGCCGAA-AGGAGCATT--GCTGA-
14  TCATTTCAGCGAGTT-AACAA-CCCGTTTT-CTTCTAAGGAACTGCGTTT-GGATTTT-CCAGAGCGAAAAGGGAGCATTTTGCTGC-
15  CA-TT-CCACGAGTT-AGCAA-CACGTT---CTTCTAGGAACTGTCGTTTGGA-TATTCC-AGTACTAAATGAGATCGTA-TGCTTT-
16  CA--TTCAGCGAGTT-AGCAA-CACGTTT--CTTCTA-GGAACTGCGTTT-GGATTTTCC-AGAGCGAAA-GGGAGCATTT-GCTGT-
17  CA--TTCAGCGTGT--AGCT--CACGTTT--CT-CTA-GGAACTGCGTTT-GGATTT--C-AGAGCTAAA-GGGGGCATTT-GCTCT-
18  CA-TTTCAGTCAGTT-AGCAA-CACGTTT-CTTCTTGGGAACTG-CGTTTGGGATATTCC-AGTGCGAAA-GGGAGCGTA-TGCTGT-
19  CA-TT-CAGCGAGTT-AGCAA-CATGTTT--CTTCTAGGAACTG-CGTTTGGA-TATTCC-AGTGCGAAA-GGGAGTGTA-TGCTGT-
20  CA--TTCAGCGAGTT-AGCAA-CACGTTT--CTTCTA-GGAACTGCGTTT-GGATTTTCC-AGAGCGAAA-GGCAGCATTT-GCTGT-
21  CA--TTCAGCGAGTTTAGCAA-CACGTTT--CTTCTA-GGAACTGCGTTT-GGATTTTCC-AGAGCGAAA-GGGAGCATTT-GCTGT-
22  CA-TT-CAGCGAGTT-AGCAA-CACGTTT--CTTCTAGGAA-CTGCGTTT-GGATTTT-CCAGAGCGAAA-GGCAGCATTT-GCTGT-
23  CA-TT-CAGCGAGTT-AGCAA-CACGTTT--CTTCTAGGAACTG-CGTTTGTA-TATTCC-AGTGCGAAA-GGGAGCGTATTGCTGT-
24  CA-TT-CAGCGAGTT-AGCAA-CACGTTT--CTTCTAGGAACTG-CGTTTGGAATATTCC-AGTGCGAAA-GGGAGCGTA-TGCTGT-
25  CA-TT-CAGCGAGTT-AGCAA-CACGATT--CTTCTACGAACTG-CGTTTGGA-TATTCC-AGTGCGAAA-GGGAGCGTA-TGCTGT-
26  CA-TT-CAGCGAGTT-AGCAA-CACGTTT--CTTCTAGGAACTG-CGTTTGGA-TATTCC-AGTGCGAAA-GGGAGCGTA-TGCTGT-
27  CA--TTCAGCGAGTT-AGCAA-CACGTTT--CTTCTA-GGAACTGCGTTT-GGATTTTCC-AGAGCGAAA-GGCAGCATTT-GCTGT-
28  CA--TTCAGCGAGCT-AGCAA-CACGTTT--CTTCTA-GGAACTGCGTTT-GGATTTTCC-AGAGCGAAA-GGCAGCATTT-GCTGT-
29  CA--TTCAGCGAGTT-AGCAA-CACGTTT--CTTCTA-GGAACTGCGTTT-GGATTTTCC-AGAGCGAAA-GGCAGCATTT-GCTGT-
30  CA--TTCAGCGAGTT-AGCAA-CACGTTT--CTTCTA-GGAACTGCGTTT-GGATTTTCC-AGAGCGAAA-GGCAGCATTT-GCTGT-
31  CA-TT-CAGCGAGTT-AGCAA-CACGTTT--CTTCTAGGAACTGCGTTTTGGA-TATTCC-GGTGCGAAA-GAGATCGTA-TGCTGT-
32  CA-TT-CAGCGAGTT-AGCAA-CACGTTT--CTTCTAGGAACTG-CGTTTGGA-TATTCC-AGTGCGAAA-GAGATCCTA-TGCTGT-
33  TCATTTCAGCGAGTT-AGCAG-CACGTTT--CTTCTA-GGAACTGCGTTTTGGATTTTCCCAGAGCGAAAAGGCAGCATTT-GCTGT-
34  CA--TTCAGCGAGTT-AGCAA-CACGTTT--CTTCTA-GGAACTGCGTTT-GGATTTTCC-AGAGCGAAA-GGGAGCATTT-GCTGT-
35  CA-TT-CAGCGAGTT-AGCAA-CACGTTTT-CTTCTAGGTA-CTGCGTTT-GGATTTC-CCAGAGCGAAA-GACAGCATTTTGCTGT-
36  CA--TTCAGCGAGTC-AGCAA-CACGTTT--CTTCTA-GGAACTTCGTTT-GGATTTTCC-AGAGCGAAA-ATGAGCATAT-GCTGT-
37  CA-TT-CAGCGAGTT-AGCAA-CACGTTTTCTTCCTAGGAACTG-CCTTTCGA-TATTCC-AGTGTGAAA-GGTAGCGTAATGCTGT-
38  CA--TTCAGCGAGTT-AGCAA-CACGTTT--CTTCTA-GGAACTGCGTTT-GGATTTTCC-AGAGCGAAA-GGCAGCATTT-GCAGT-
39  CA--TTCAGCGAGTT-AGCAA-CACGTTTC-T-TCTAGGAACT-GTGTTT-GCATTTTCC-AGAGCGAAA-GGGGGCATTT-GCTGT-
40  CA--TTTAGCGAGTT-AGCAA-CACGATT--ATTCCC-GGAACTGCGTTT-GGATTTTCC-AGAGCGAAA-GGGAGCATTT-GCTGT-
41  CA-TT-CATCGAGTT-AGCAA-CACCTTT--CTTCTAGGAACTG-CGTTTGGA-TATTCC-AGTGCGAAA-GGGAGCGTA-TGCTGT-
```

Figure S1, page 8 of 12

Squ (n=40), 1 of 2

```
              .........+.........+.........+.........+.........+.........+.........+.........+.........+.........1
          Con AAAGAGAAA-TACCTT--GCT-CTAAAA-CCAAAACG-GAGCTATCTAGCAGAA-TGGTTGTCAAT-GTG-TGTATTC--AACTTCCAGAGTT-AAACTG
Squ106a   01  AAAGAGAAT-TACCTT--GCT-CTAAAACC-AAAACG-GAGCTATCTAGCAGAA-TGGTCGTCAAT-GTG-TGTTTTC--AACTTCCAGAGTT-GAACTG
Squ107a   02  AAAGAGAAA-AACCTT--GTT-CTAAAA-CCAAAACG-GAGCTATCTAGCAGAA-TGTTTGTCAAA-GTG-TGTATTC--AACTTCCATAGTT-AAACTG
Squ107b   03  AAAGAGAAA-AACCTT--GCT-CTAAAA-CCAAAACG-GAGCTATCTAGCAGAA-TGTTTGTCAAA-GTG-TGTATTC--AACTTCCAGAGTT-AAACTG
Squ109a   04  AAAGAGAAA-TACCTT--GCT-CTAAAA-CCAAAACG-GAGCTATCTAGCAGAA-TGGTTGTCAAT-GTG-TGTATTC--AACTTCCAGAGTT-AAACTG
Squ109b   05  AAAGAGAAA-TACCTT--GCT-CTAAAA-CCAAAACG-GAGCTATCTAGCAGAA-TGGTTGTCAAT-GTG-TGTATTC--AACTTCCAGAGTT-AAACTG
Squ110a   06  AAAGAGAAA-TACCTT--GCT-CTAAAA-CCAAAACG-GAGCTATCTAGCAGAA-TGCTTGTCAAT-GTG-TGCATTC--AACTTCCAGAGTT-AAACTG
Squ110b   07  AAAGAGAAA-TACCTT--GCT-CTAAAA-CCAAAACG-GAGCTATCTAGCAGAA-TGGTTGTCAAT-GTG-TGCATTC--AACTTCCAGAGTT-AAACTG
Squ111a   08  AAAGAGAAA-TACGTT--GCT-CTAAAA-CCAAAACG-GAGCTATCTAGCAGAA-TGGTTGTCAAT-GCG-TTTATTC--AAGTTCCAGAGTT-AAACTG
Squ111b   09  AAAGAGAAA-TACCTT--GCT-CTAAAA-CCAAAACG-GAGCTATCTAGCAGAA-TGGTTGTCAAT-GCG-TTTATTC--AAGTTCCAGAGCT-AAACTG
Squ01G7a  10  AAAGAGAAA-TACCTT--GCT-ATAAAA-CCAAATCG-GAACTGTCTAGCAG-AATGGTTGTCAAC-GCG-TGTATTC--AACTTCCAGAGTT-AAACTC
Squ02K5a  11  AAAGAGAAA-TACATT--GCT-CTAAAA-CCAAAACGGAG-CTATCTAGCAGAA-TGGTTGTCAAT-GTG-TGTATTC--AACTTCCAGAGTT-AAACTG
Squ02L6a  12  AAAGTGAAA-TACCTT--GCT-CTAAA--CCTAAACG-GAGCTATCTAGCAGAA-TGTTTGTCAAA-GTG-T--ATTC--AACTTCCAGAGTT-AAACAG
Squ05B8a  13  AAAGAGAAA-TACCTT--GCT-CTAAAA-CCAAAACG-GAGCTATCTAGCAGAA-TGGTTGTCAAT-GTG-TGTATTC--AACAGCCAGAGTT-AAACTG
Squ05B8b  14  AAAGAGAAA-TACCTT--GCT-CTAAAA-CCAAAACG-GAGCTATCTAGCAGAA-TGGTTGTCAAT-CTG-TGTATTC--AACTTCCTGAGAT-TAACTG
Squ07I7a  15  AAAGAGAAA-AACCTT--GCT-CTAAAA-CAAAAACG-GAGCTATCTAGCAGAA-TGTTTTTCAAT-GTC-TTTATTC--AACTTCCAGAGTT-AAACTG
Squ08A5a  16  AAAGAGAAG-TACCTT--GCT-GTAAAA-CAAAAACG-GAGCTATCTAGCAG-AATGGTTGTCAAT-GTG-TGCATTC--ATCTTCCAGAGCT-AAACTG
Squ09D5a  17  AAAGTGAAA-TACCTT--GCT-CTAAAA-CCGAAACG-GAGCTATCTAGCAGAA-TGGTTGTCAAT-GTG-TGTATAC--AACTTCCAGAGTT-AAACTG
Squ10H4a  18  AAAGAGAAG-TACCTT--GCC-CTAAAA-CAAAATCG-GAGCTATCTTGCAGAA-TGGTTGTCAAT-GTG-TGCATTC--AACTTCCAGAGTT-AAACTG
Squ10K5a  19  AAAGAGAAG-TACCTT--GCT-TTAAAA-CAAAAAAGTAAGCTACCTAGCAGGAATGGTTGTCAAT-GTA-TTCCTTTCAGCTTTCCATAGTT-AATCTG
Squ31G4a  20  -AAGAGAAA-TACCTT--GCT-CTAAAA-CCAAAACG-GAGCTATGTAGCAGAA-TGGTTGGCAAT-GTG-TGTATTC--AACTTCCAGAGTT-AAACTG
Squ32J5a  21  AAAGAGAAA-TATTTT--GTC--TAAAA-CCAAAACG-GAGCTATCTAGCAG-AATGTCTGTCAAT-GTG-TGTATTC--AACTTCTAGAGAT-AAACTC
Squ35C3a  22  AAAGAGAAAATACCTT--GCT-CTAAAACCCAAAACG-GAGCTATCTAGCAGAA-TGGTTGCCAAT-GTG-TGTATTC--AACTTCCAGAATT-AACCTG
Squ35C3b  23  AAAGAGAAA-TACCTT--GCT-CTAAAACC-AAAACG-GAGCTATCTAGCGGAA-TGGCTGTCAAT-GTG-TGTATTC--AACTTACAGAGTT-TAACTG
Squ35D1a  24  AAAGAGAAA-TACCTT--GCT-CTTAAAACCAAAACG-GAGCTATCTAGCAGAA-TGGTTGCCAAT-GTG-TGTATTC--AACTTCCAGAATT-AACCTG
Squ35D1b  25  AAAGAGAAA-TACCTT--GCT-CTAAAACC-AAAACG-GAGCTATCTAGCGGAA-TGGCTGTCAAT-GTG-TGTATTC--AACTTACAGAGTT-TAACTG
Squ36G2a  26  AAGGAGAAA-TACCTT--GCT-CTAAAA-CGAAAACGGAG-CTATCTAGCAGAA-TGGTTGTCAAT-GTG-TGTATTC--AACTTCCAGAGTT-AAACTG
Squ36G2b  27  AAAGAGAAA-TACCTT--GCT-CTAAAA-CCAAAACG-GAGCTATCTAGCAGAA-TGGTTGTCAAT-GTG-TGTATTC--AACTTCCAGAGTT-TAACTG
Squ38E1a  28  AAAGAGAAA-TACCCT--GCT-CTAAGC-C--AAACG-GAGCTATAGTGCAGAA-TGGTTGTCAAT-GTG-TGTATTC--AACTTCCAGAGAT-AAACTG
Squ38E1b  29  AAAGAGAAA-TACCTT--GCT-CTAAAA-CCAAAACG-GAGCTATCTAGCAGAA-TGGTTGTCAAT-GTG-TGTATTC--AACTTCCAGAGTT-AAACGG
Squ39B4a  30  AAAGAGTAA-TACCTT--GCT-CTAAAA-CCAAAACG-GAGCTATCTAGCAGAA-TGGTTGTCAAT-GTG-TGTATTC--AACTTCCAGAGTT-TAACTG
Squ39B4b  31  AAAGAGAAA-TACCTT--CCT-CTAAAA-CCAAAACG-GAGCTATCTAGCAGAA-TGTTTGTCAAT-GTG-TGTATTC--AACTTCCAGAGTT-AA-CTG
Squ39L6a  32  -AAGAGAAA-TACCTT--GCT-CTAAAA-CCAAAACGAGAGCTATCTAGCAGAA-TGGTAGTCAAT-GTG-TGTATTC--AACTTCCAGAGTTTAAACTG
Squ39L6b  33  AAAGAGAAA-TACCTT--GCT-CTAAAA-CCAAAACG-GAGCTATCTAGCAGAA-TGGTTGTCAAT-GTG-TGTATTC--AACTTCCAGAGTT-AAACGG
Squ40B6a  34  AAGGAGAAT-TAACTCTGGCTGCTAAAATCCAAAACGGAGGCTATCTAGCAGAA-TGGTTGTCAATTGTGGTGTATCA--ACTTCCCAGAGTTTAAACTG
Squ40B6b  35  AAAGAGAAA-TACCTT--GCT-CTAAAT-CCAAAACG-GAGCTATCTAGTAGAA-TGGTTGTCAAT-GTG-TGTATTC--AACTTCCAGAGAT-AAACTG
Squ41C3a  36  AAAGAGAAA-TACCTT--GTT-CTAAAACC-AAAACG-GAGCTATCTAGCAGAA-TGGTTGTCAAT-GTG-TGTATTC--AACTTCCAGATTT-AAACTG
Squ41L7a  37  AAAGAGAAAATACCTT--GCT-CTAAAACC-AGAACG-GAGCTATCTAGCAGAAATGGTTGCCAAT-GTG-TGTATTC--AACTTCCAGAATT-AACATG
Squ41L7b  38  AAAGAGAAA-TACCTT--GCT-CTAAAACC-AAAACG-GAGCTATCTAGCGGAA-TGGCTGTCAAT-GTG-TGTATTC--AACTTACAGAGTT-TAACTG
Squ41L8a  39  AAAGAGAAA-TACCTT--GCT-CTAAAACCAAAAACG-GAGCTATCTAGCAGAA-TGGTTGCCAAT-GTG-TGTATTC--AACTTCCAGAATT-AAC-TG
Squ41L8b  40  AAAGAGAAA-TACCTT--GCT-CTAAAACC-AAAACG-GAGCTATCTAGCGGAA-TGGCTGTCAAT-GTG-TGTATTC--AACTTACAGAGTT-TAACTG

              .........+.........+.........+.........+.........+.........+.........+.........+.........+.........2
          Con ATGTG-TGTTTGCAGCAGTT-CAGAAA-CCCTTTCTTTGGAGAA-TGTA-GAAACAGTCATTTCCAGCCCTAGAGACGCAT-ATAAGA-GTTAGCAGTAA
          01  ATGTG-AGTTTGCAGCAGTT-CAGAAA-CCCTTTCTTTGGAGAA-TGCAGAAA-CAGTCATTTCCAGATCTAGAGACGCAT-ATAA-GAGTTAGCAGTAA
          02  ATGTT-TGTTTGCAGCAGTT-CAGAAA-CCCTTTCGTTGGAGAA-TGTA-GAAACAGTCATTTCCAGCCCTAGAAACGCAT-GTAAGA-GTTAGCAGTAA
          03  ATGTT-TGTTTGCAGCAGTT-CAGAAA-CCCTTTCGTTGGAGAA-TGTA-GAAACAGTCATTTCCAGCCCTAGAAACGCAT-GTAAGT-GTTAGCAGTAA
          04  ATGTG-TGTTTGCAGCAGTT-CAGAAA-CCCTTTCCTGGGAGAA-TGTA-GAAACAGTCATTTCCAGCCCTAGAGACGCAT-ATAAGA-GTTAGCAGTAA
          05  ATGTG-TGTTTGCAGCAGTT-CAGAAA-CCCTTTCCTGGGAGAA-TGTA-GAAACAGTCATTTCCAGCCCTAGAGACGCAT-ATAAGA-GTTTGCAGTAA
          06  ATGTG-TGTTTGCAGCAATT-CAGAAACCCTTT-CTTTGGAGAA-TGAA-GAAACGGTTATTTACAGCCCCAGAGACGCTT-ATAAGAGTT-AGCGGTAA
          07  ATGTG-TCTTTGCAGCAGTT-CAGAAACCCTTTTCTTTGGAGAA-TGAA-GAAACAGTTATTTCCAGCCCTATAGAGGCTTTATAAGAGTT-AGCAGTAA
          08  CTGTG-TGTTTGCAGCAGTT-CAGAAACCTTTT-CTTTGGAGAA-TGCA-GAAACTGTCTTTTCCTGCCCTAGAGACGCTT-ATAAGTGTT-AGCAGTAA
          09  CTGTG-TGTTTGCAGCAGTT-CAGAAACCCTTT-CTTTGGAGAC-TGCA-GAAAAAGTCTTTTCCCGCCCCAGAGACGCTT-AGGAGAGTTTAGCAGTAA
          10  ATGTT-TGTTTACAGCAGTT-CAGAAA-CCCTTTCTTTGGAGAA-TGTA-GAAACAGTCATTTCCAGCCCTAGAGACGCAT-ATAAGA-GTGAGCAGTAA
          11  ATGTG-TGTTTGCAGCAGTT-CAGAAA-CCCTTTCCTGGGAGAA-TGT-AGAAACAGTCATTTCCAGCCCTAGAGACGCAT-ATAA-GAGTTTGCATTAA
          12  ATGTC-TGTTTGCAGCAGTT-CAGAAA-CCGTTTCTAGGGAGAA-TGTA-GAAACAGTCATTTCCAGCCCTAGAGACGCAT-ATAAAG-GTTTGCAGTAT
          13  ATGTG-TGTTTGCAGCAGGT-TAGAAA-CCCTTTCTTTGGAGAA-TGCA-GAATTAGTCATTTCAAGCCCTTGAGACGCAT-ATAAGA-GTTAGCAGTAA
          14  ATTTG-TGTTTGCAGCAATT-CAGAAACCCTTTCCTTTGGGAGAATGCAGAAACCGTCCATTTCAAGCCCTAGAGACACAT-ATAAGAGTTTAGCACTAA
          15  ATGTG-TGTTTGCAGCAGTT-CAGAAA-CCCTTTCTTTGGAGAA-TGTA-GAAACAGTCATTTCCAGCCCTAGAAACGCAT-GTAAGA-GTAAGCAGTAA
          16  CTGTG-TGTTTGCAGCAGTT-CAGAAA-ACCTTTCTTTGGAGAA-TGTA-GAAACAGTCATTTCCAGCCCCAGAGACGCAT-GTAAGA-GATAGTAGTAA
          17  TTGTG-TGTTTGCAGCAGCT-CAGAAA-CACTTTCCTTGGAGAA-TGTA-GAAACAGTCATTTCCAGCCCTAGAGACGGAT-ATAAGT-GTTATCAGTAA
          18  ATGTG-TGTTTGCAGCAGTT-CAGAAA-CCCTTTCTTTGGAGAA-TGTA-GAAATAGTCCTTTCCAGCCCTAGAGACGCAT-ATAAGA-GTTAGCAGTAA
          19  ATGTG-TGTTTGCAGCCGTTTCAGAAA-CCCTTTCTTTGGAGAA-TGTA-GAAACAGTCATTTCCAGCCCTAGAGACACAT-ATAAGA-GTTAGCAGTAA
          20  ATGTG-TGTTTGCCGCAGCT-TGGAAA-CCCTTTCTTTGGAGAA-TGCA-GAAACAGTCATTTCCAGCCCTAGAGACGGAT-ATAAGA-GTTAGCAGTAA
          21  ACGTG-TGTTTGCAGCAGTT-CAGAAA-CCCTTTCTTTGGAGAA-TGTA-GAAACAGTCATTTCCAGACCTAGAGACGCAT-ATAAGA-GTTAGCAGTAA
          22  ATGTG-TGTTTGCAGCAGTT-CAGAAA-CCCTTTCTTTGGAGAA-TGCAGAAA-CAGTCATTTCCAGCCCTAGAGACGCAT-ATAA-GAGTTAGCAGTAA
          23  ATGTG-TGTTTGCAGCAGTT-CAGAAA-CCCTTTCTTTGAACAA-TGCAGAAA-CAGTCATTTTCAGCCCTAGAGACGCAT-ATGA-GAGGTAGCAGTAA
          24  ATGTG-TGTTTGCAGCAGTT-CAGAAA-CCCTTTCTTTGGAGAA-TGCAGAAAACAGTCATTTCCAGCCCTAGAGACGCAT-ATAAAGAGTTAGCAGTAA
          25  ATGTG-TGTTTGCAGCAGTT-CAGAAA-CCCTTTCTTTGAACAA-TGCAGAAA-CAGTCATTTTCAGCCCTAGAGACGCAT-ATGA-GAGGTAGCAGTAA
          26  ATGTG-TGTTTGCAGCAGTT-CAGAAA-CCCTTTCCTGGGAGAA-TGT-AGAAACAGTCATTTCCAGCCCTAGAGACGCAT-ATAA-GAGTTTGCAGTAA
          27  ATGTG-TGTTTGCAGCAGTT-CAGAAA-CTCTTTCCTGGGAGAA-TGTA-GAAACAGTCATTTCCAGCCCTAGAGACGCAT-ATTAGG-GTTAGCAGTAA
          28  ATGTG-TGTTTGCAGCAGTT-CAGAAA-CCCTTTCCTGGGAGAA-TGTA-GAAACAGTCATTTCCACCCCTAGAGACGCAT-ATAAGA-GTTTGCAGTAA
          29  ATGTG-TGTTTGCAGCAGTT-CAGAAA-CCCTTTCCTGGGAGAA-TGTA-GAAACAGTCATTTCCAGCCCTAGAGACGCAT-ATAAGA-GTTTGCAGTAA
          30  ATGTG-TGTTTGCAGCAGTT-CAGAAA-CCCTTTCCTGGGAGAA-TGTA-GAAACAGTCATTTCCAGCCCTAGAGACGCAT--AAAAGAGTTTGCAGTAA
          31  ATGTTGTGTTTGCAGCAGTTTCAGAAA-CCTTTTCCTGGGAGAAATGTAAGAAACAGTCATTTCCACCCTTAGAGACGCATTATAAAAAGTTTGCAGTAA
          32  ATGTG-TGTTTGCAGCAGT--CAGAAA-CCCTTTCCTGGGAGAA-TGTA-GAAACAGTCATTTCCAGCCCTAGAGACGCAT-ATAAGA-GTTTGCAGTAA
          33  ATGTG-TGTTTGCAGCAGTT-CAGAAA-CCCTTTCCTGGGAGAA-TGTA-GAAACAGTCATTTCCAGCCCTAGAGACGCAT-ATAAGA-GTTTGCAGTAA
          34  ATGTG-TGTT-GCAGCAGTT-CAGAAAACCCTTTCCTGGGAGAA-TGTTAGAAACAGTCATTTCCAGCCCTAGAGACGCAT-ATAAAGAGTTTGCAGTAA
          35  ATGTA-TGTTTGCCGCAGTT-CAGAAA-CCCTTTCCTGGGAGAA-TGTA-GAAACAGTCATTTCTAGCCCTAGAGACGCAT-ATAAGA-GTTAGCAGTAA
          36  ATGTG-TGTTTGCAGCAGTT-CTGAAA-CCCTTTCTTTGGAGAA-TGTAGAAA-CAGCCGTTTCCAGATCTAGAGACGCAT-GTAA-GAGTTAGCAGTAA
          37  ATGTG-TGTTTGCAGCAGTT-CAGAAA-CCCTTTCTTTGGAGAA-TGCAGAAA-CAGTCATTTCCAGCCCTAGAGACGCAT-ATAA-GAGTTAGCAGTAA
          38  ATGTG-TGTTTGCAGCAGTT-CAGAAA-CCCTTTCTTTGAACAA-TGCAGAAA-CAGTCATTTTCAGCCCTAGAGACGCAT-ATGA-GAGGTAGCAGTAA
          39  ATGTG-TGTTTGCAGCAGTT-CAGAAA-CCCTTTCTTTGGAGAA-TGCAGAAA-CAGTCATTTCCAGCCCTAGAGACGCAT-ATAA-GAGTTAGCAGTAA
          40  ATGTG-TGTTTGCAGCAGTT-CAGAAA-CCCTTTCTTTGAACAA-TGCAGAAA-CAGTCATTTTCAGCCCTAGAGACGCAT-ATGA-GAGGTAGCAGTAA
```

Figure S1, page 9 of 12

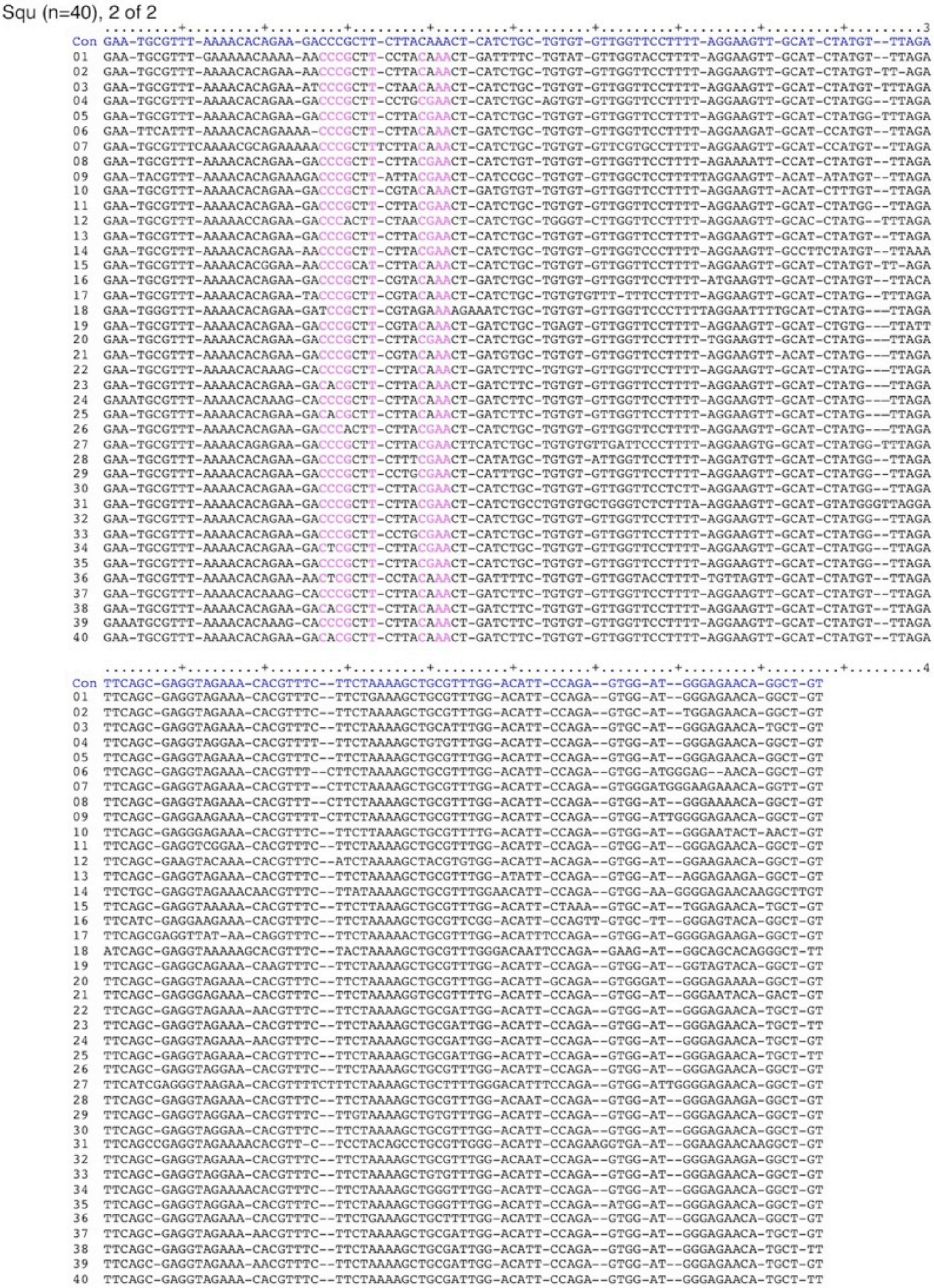

Figure S1, page 10 of 12

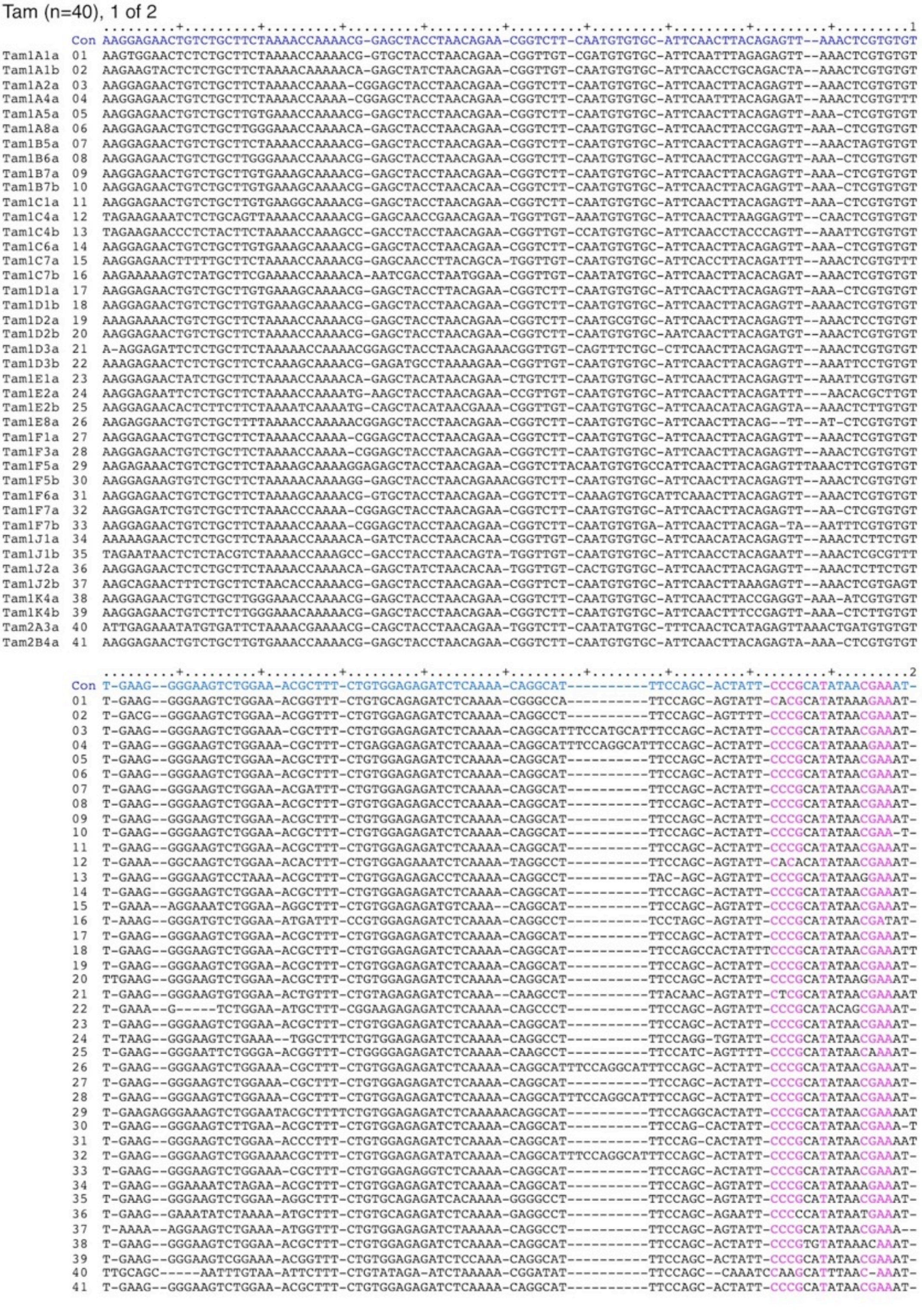

Figure S1, page 11 of 12

Tam (n=40), 2 of 2

```
    .........+.........+.........+.........+.........+.........+.........+.........+.........+.........3
Con AGCAGTAAAA-CTCTGCCTAAAACACAGAAGAAA-CGTATCCTTCA-ATCTGCTCTGCTGTGCGTTTGTTC-AGCT-AAGTG-AGTT-AAATCTC-CCTT
01  AGCAGTAAAA-CTCTGCCTAAAACAAAGAAGAAA-CGTATCTTTCA-GTCTGCTCTGCTGTGCGTTCGTTC-AGCT-AAGTG-AGTT-AAATATC-CCTT
02  AGCAGTAAAA-CTCTGCCTAAAACACAGAAGAAA-CGTATCCTTCA-ATCTGCACTGCTGTGCCTTTGTTC-AGCT-AAGTG-AGTT-AAATCTC-CCTT
03  AGCAGTAAAA-CTCTGCCTAAAACATAGAAGAAA-CGTATCCTTCA-ATCTGCTCTGCTGTGCGTTTGTTC-AGCT-AAGTG-AGTT-AAATCTC-CCTT
04  AGCAGTAAAA-CTCTGCCTAAAACACAGAAGAAA-CGTATCCTTCA-ATCTGCTCTGCTGTGCGTTTGTTC-AGCT-AAGTG-AGTTTAAATCTC-CCTT
05  ATCAGTAAAA-CTCTGCCTAAAACACAGAAGCAA-CGTATCCTTCA-ATCTGCTCTGCTGTGCGTTTGTTC-AGCT-AAGTG-AGTT-AAATCTC-CCTT
06  AGCAGTAAAA-CTCTGCCTAAAACACAGAAGCAA-CGTATCCTTTA-ATCTGCTCTGCTGTGCGTTTGTTC-AGCT-AAGTG-AGTT-AAATCTC-CCTT
07  AGCAGTAAAA-CTCTGTCTAAAACACAGAAGAAA-CGTATCCTTCA-ATCTGCTCTGCTGTGCGTTTGTTC-AGCT-AAGTG-AGTT-AAATCTCCCTTT
08  AGCAGTAAAA-CTCTGCCTAAAACACAGAAGCAA-CGTATCCTTCA-ATCTGCTCTGCTGTGCGTTTGTTC-AGCT-AAGTG-AGTT-AAAACTC-AGTT
09  ACCAGTAAAA-CTCTGCCTAAAACACAGAAGCAA-CGTATCTTTCA-ATCTGCTGTGCTGTGCGTTTGTTC-AGCT-AAGTG-AGTT-AAATGTC-CCTG
10  AGCAGTAAAA-CTCTGCCTAAAACACAGAAGCAA-CGTATCCTTCA-ATCTGCTCTGCTGAGCGTTTGTTTCAGCT-CAGAG-AGTT-AAATGTC-CCTG
11  AGCAGTAAAA-CTCTGCCTAAAACACAGAAGCAA-CGTATCCTTCA-ATCTGCTCTGCTGTGCGTTTGTTC-AGCT-AAGTG-AGTT-AAATGTC-CCTG
12  AGCAGTAAAA-CTCTGCCTAAAACACAAAAGC-AACCTATCTTTCA-AACTGCTCTGCTGTGC-----------T-AAGTG-AGTAAAA--TCTCCCTT
13  AGCAGTAAAAACTCTGCCTAAAACACAGAAACCAACGTATCCTTCT-ATCTTCTCTCCTGTGCGTTTGTTC-AGCT-AAGGG-AGTTAAAATCTCCCCTT
14  AGCAGTAAAA-CTCTGCCTAAAACACAGAAGCAA-CGTATCCTTCA-ATCTGCTCTGCTGTGCGTTTGTTC-AGCT-AAGTG-AGTT-AAATGTC-CCTG
15  ACCAGTAAAA-TTTTGCCTAAAACACAGAAGC-AACGTATCCATCA-ATCTGCTCTGGTGTTCCTTTATTC-AGCT-AAATG-AGTTAAA--TCTCCCTT
16  AGCAGTAAAA-CTCTGCTTAAAACACAGAAGG-AAGGTATCCTTCA-ATCTGCTCTGCTGTGCATTTCTTA-TGCT-AAGTG-TGTTAAC--TCTCACTT
17  AGCAGTAAAA-CTCTGCCTAAAACACAGAAGCAA-CGTATCCTTCA-ATCTGCTCTGCTGTGCGTTTGTTC-AGCT-AAGTG-AGTT-AAATGTC-CCTG
18  AGCAGTAAAA-CTCTGCCTAAAACACAGAAGCA--CGTATCCTTCA-ATCTGCTCTGCTGTGCGTTTGTTCCAGCTTAAGTGGAGTT-AAATGTCTCCTG
19  AGCAGTAAAA-CTCTGCCTGAAACACAGAAGAAA-CGTATCCTTCA-ATCTGCTCTGCTGTGCGTTTGTTC-AGCT-AAGTG-AGTT-AAATCTC-CCTT
20  AGCAGTAAAA-CTCTGCCTGAAACACAGAAGAAA-CGTATCCTTCA-ATCTGCTCTGCTGTGCGTTTGTTC-AGCT-AAGTG-AGTT-AAATCTC-CCTT
21  AGCAGTAAAA-CTCTGCCTAAAACACAGAAGCAA-CGTATCTTTCA-ATCTCCTCTGCTGTTCATTTGTTC-AGCT-ATGTC-AGGTAAA--TCTCCCTT
22  AGCAGTAAAA-CTCTTCCTTAAACACAGAAGG-AAAGTATACTTCA-ATCTGCTCTGCTGTTCCTTTGTTC-AGCT-AAGTG-AGTTAAA--TCTCCATT
23  AGCAGTATAA-CTCTGCCTAAAACCCAGAAGAAAACGTATCCTTCA-ATCTGCTCTGCTGTGCGTTTGTTTCAGCT-AAGTG-AGTT-AAATCTCCCTTT
24  CGCATTAAAA-CTCTGCGTAAAACACCGAGGAAA-CGTATCATTCA-ACTTTCTCTGCTGTGCCTCTGTTC-AGCT-AACTG-AGTTAAA--TCGCCTTT
25  ACCAGTAAAA-CTCTGTCTAAAACACAGAAGAAA-CGTATTCTACACACCTGCTATGCAGTGCCTTTGTTC-AGCT-AAATG-AATTGAGTCTCTCCTTT
26  AGCAGTAAAA-CTCTGCCTAAAACACAGAAGAAA-CGTATCCTTCA-ATCTGCTCTGCTGTGCGTTTGTTC-AGCT-AAGTG-AGTT-AAATCTC-CCTT
27  AGCAGTAAAA-CTCTGCCTAAAACACAGAAGAAA-CGTATCCTTCA-ATCTGCTCTGCTGTGCGTTTGTTC-AGCT-AAGTG-AGTT-AAATCTC-CCTT
28  AGCAGTAAAA-CTCTGCCTAAAACACAGAAGAAA-CGTATCCTTCA-ATCTGCTCTGCTGTGCGTTTGTTC-AGCT-AAGTG-AGTT-AAATCTC-CCTT
29  AGCAGTAAAAACTCTGCCTAAAACACAGAAGAAA-CGTATCATTCA-ATCTGCTCTGCTGTGCGTTTGTTC-AGCT-AAGTG-AGTT-AAGTCTC-CCTT
30  AGCAGTAAAA-CTCTGCCTAAAACACAGAAGAAA-CGTATCCTTCA-ATCTGCTCTGCTGTGAGTTTGTTC-AGCT-AAGTG-AGTT-AAGTCTC-CCTT
31  AGCAGTAAAA-CTCTGCCTAAAACACAGAAGAAA-CGTATCATTCA-ATCTGCTCTGCTGTGCGTTTGTTC-AGCT-AAGTG-AGTT-AACTCTC-CCTT
32  AGCACTAAAA-CTCTGCTTAACACACAGAAGAAA-CGTATCCT-CA-ATCTGCTCTGCTGTGCGTTTGTTC-AGCT-AAGTG-AGTT-AAATCTC-CCTT
33  AGCAGTAAAA-CTCTGCCTAAAACACAGAAGAAA-CGTATCCTTCA-ATCTGCTCTGCTGTGCGTTTGTTC-AGCT-AAGTG-AGTT-AAATCTC-CCTT
34  AGAAGTATAA-CTCTGCCAAAAACACAGAAGC-AGCGTATCATTCA-ATCTGTTCTGGTGTGCCTTTATTC-AGCG-AAGTG-ACTTAAA--TCTCCCTT
35  AGCAGTATAA-CTCTGCCTAAAACACAGAAGG-AAGGTATCCTTCA-ATCTGCTCTGCTGTGCATTTCTTA-TGCT-AAGTG-TGTTAAA--TCTCCCTT
36  AGCAGTAAAA-CTCGGCCTAAAAGACAGAAGC-AACGTATCATTCA-ATCTGCTCTGCTGTGCGTAAGTTC-AGCT-AAGTG-AGTTAAA--TCTCCATC
37  AGCAGTATAA-TTCTGCCTTAAACACAGAAGC-AACATATCCTTCA-ATCTGCTCAGCTGTGCGTTTGTTC-TGCT-AAGTG-AGTTAAA--TCTCCCTT
38  AGCAGTAAAA-CTCTGCCTAAAACACAGAAGGAA-CGTATCCTTCA-ATCTGCTCTGCTTTGCGTTTGTTC-AGCT-ATGTG-AGTT-TAATCTC-CCTT
39  TGCAGGAAAA-CTCTGCCTAAAACACAGAAGCAA-CGTATCCTTCA-ATCTGCTCTGCTGTGCGTTTGTTC-AGCT-AAGTG-AGTT-AAATCTC-CCTT
40  AGCTGTAAGA-CTGCATCTAAAACACACAAAAAA-CGTATCTTTCA-TACTGCTCTCATGTGTGTTCGTTTAACAT--AGAG-AGTTGAA--TGTATGTC
41  AGCAGTAAAA-CTCTGCCTAAAACACAGAAGCAA-CGTATCCTTCA-AACTGCTCTGCTGTGCGTTTGTTC-AGCT-AAGTG-AGTT-AAATCTC-CCTT

    .........+.........+.........+.........+.........+.........+.........+.........+.........+.........4
Con TAGATTC-AGGAGTTAGAAA-CAC-GTTTCTGGTAAGA-GCTGCGTTTGGA-CATTT-CTCAGTGAA-TGGGAGTAT-ATGC-TGT
01  TAGATTT-AGGAGTTAGAAA-CACGTTT-CTGGTAATA-GCTGAGATTATA-CATTC-CTGAGTGAA-TGAGAGTAT-ATGT-TGT
02  TAGATTT-AGGAGTTAGAAT-CACGTTTCTGGTAAGA-GCGGAATTTTTA-CATTC-CGCAGTGAG-TGGCAGTCT-GTGC-TGT
03  TAGATTC-AGGAGTTAGAAA-CAA-GTTTCTGGTAAGA-GCTGCGTTTGGA-CATTT-CTCAGTGAA-TGGGAGAAT-ATGT-TGT
04  TAGATTC-AGGAGTTAGAAA-CAC-GTTTCTGGTAAGA-GCTGCGTTTGGA-CATTT-CTCAGTGAA-TGGGAGTAT-ATGC-TGT
05  TAGATTC-AGGAGTTAGAAA-CAC-GTTTCTGGTAAGA-GCTGCGTTTGGA-CATTT-CTCAGTGAA-TGGGAGCCT-ATGC-TGT
06  TAGATTC-AGGAGTTAGAAA-CAC-GTTTCTGCTAAGA-GCTGCGTTTGGA-CATTT-CTCAGTAAA-TGGGAGCCT-ATGC-TGT
07  TAGATTC-AGGAGTGAGAAA-CAC-GTTTCTGGTAAGA-GCTGCGTTTGGACATTTT-CTCAGTGAA-TGGGAGTAT-ATGA-TGT
08  TAGATTC-AGGAGTTAGAAA-CAC-GTTTCTGCTAAGA-GCTGCCTTTGGA-CATTT-CTCAGTGAA-TGGGTGCCT-ATGC-TGT
09  TAGATTC-AGGAGTTAGAAA-CAC-GTTTCTGCTAAGA-GCTGCGTTTGGA-CATTT-CTCAGTGAA-TGGGAGCCT-ATGC-TGT
10  TAGATTC-CGGAGTTAGAAA-CAC-GTTTCTGCTAAGA-GCTGCATATTGGACATTT-CTCAGTGAA-TGGGAGCCT-ATGC-TGT
11  TAGATTC-AGGAGTTAGAAA-CAC-GTTTCTGCTAAGA-GCTGCGTTTGGA-CATTT-CTCAGTGAA-TGGGAGCCT-ATGC-TGT
12  TAGATTC-ATGGGTTAAAAA-CAC-GTTTCTGGCAATG-GCTGCGTTT-GGACATTCC-TGAATGAATTGG-AGTAT-ATAC-TGT
13  TAGATTC-AGGAGATAGAAG-CAC-GTTTCTGGCAAGT-GCTGCATTTTGGACATTCCCTGAATGAATGGGGAATAT-ATGC-TGT
14  TAGATTC-AGGAGTTAGAAA-CAC-TTTTCTGCTAAGA-GCTGCGTTTGGA-CATTT-CTCAGTGAA-TGGGAGCCT-ATGC-TGT
15  TAGATTC-AGGAGTTAGAAA-CAC-ATTTCTGGCAAGG-GCTGCGTTT-GGACATTCC-TGCGTTAATGGG-AGTAT-ATGC-TGT
16  TAGATTC-AGGAGATAGAAA-CAC-GTTCCTGGCAAGT-GCTGCGTTT-GGACATTTC-TGAGTGAATGGG-AATAT-ATTC-TGT
17  TAGATTC-AGGAGTTAGAAA-CAC-GTTTCTGCTAAGA-GCTGCGTTTGGA-CATTT-CTCAGTGAA-TGGGAGCCT-ATGC-TGT
18  TAGATTCCAGGAGTTAGAAAACACATTTTCTGCTAAGAAGCTGCGTTTGGAACATTT-CTCCATGAAATGGGAGCCTTATGCCTGT
19  TAGATTC-AGGAGTTAGAAA-CAC-GTTTCTGGTAAGA-GCTGCGTTTGGA-CATTT-CTCAGTGAA-TGGGAGTCT-ATGC-TGT
20  TAGATTC-AG-AGTTAGAAA-CAC-GTTTCTGGTAAGA-GCTGCGTTTGGA-CATTT-CTCAGTGAA-TGGGAGTCT-ATGC-TGT
21  TAGATTC-AGGAGTTAGAAA-CAA-GTTTCTGGAAAGT-GCTGCATTTGGA-CATTC-CTGAGTGAA-TGGGAGAAT-ATGC-TGT
22  TAGATTC-AGGAGTTAGAAA-CAC-GTTTCTATCAAAA-GCTGGGTTA-GGACATTCC-TAAGTGAATTGG-AGTAT-ATGC-TAT
23  TAGATTTCAGGAGTTAGAAA-CAC-GTTTCTGGTAATA-GATGCGTTTTGGACATTTTCTCAGTGAA-TGGGAGGAT-ATGC-TGT
24  TAGATTC-AGGAGTTAGAAA-CAC-GTTTCTGGTAAGA-GCTG-GTTTGGA-CATTC-CTGAGTGAA-TGTGAGTTT-ATGC-TGT
25  AATAATC-AGTAGCTGGAAA--ACAGGTTCTGGTTAGA-GCTTC-TTTGGAACATTC-CTGAGTTTAATGG-AATAC-CTGC-TGT
26  TAGATTC-AGGAGTTAGAAA-CAC-GTTTCTGGTAAGA-GCTGCGTTTGGA-CATTT-CTCAGTGAA-TGGGAGTAT-ATGC-TGT
27  TAGATTC-AGGAGTTAGAAA-CAC-GTTTCTGGTAAGA-GCTGCGTTTGGA-CATTT-CTCAGTGAA-TGGGAGTAT-ATGC-TAT
28  TAGATTC-AGGAGTTAGAAA-CAC-GTTTCTGGTAAGA-GCTGCGTTTGGA-CATTT-CTCAGTGAA-TGGGAGTAT-ATGC-TGT
29  TAGATTC-AGGAGTTAGAAA-CAC-GTTTCTGCTAAGA-GCTGCGTTTGGA-CATTT-CTCAGTGAA-TGGGAGCCT-ATGC-TGT
30  TAGATTC-AGGAGTTAGAAA-CAC-GTTTCTGCTAAGA-GCTGCGTTTGGA-CATTT-CTCAGTGAA-TGGGAGCCT-ATGC-TGT
31  TACATTC-AGGAGTTAGAAA-CAC-GTTTCTGCTAAGA-GCTGCGTTTGTA-CATTT-CTCAGTGAA-TGGGAGCCT-ATGC-TGT
32  TAGATTC-AGGAGTTAGAAA-CAC-GTTTCTGGTAAGA-GCTGCGTTTGGA-CATTT-CTCAGTGAA-TGGGAGTAT-ATGC-TGT
33  TAGATTC-AGGAGTTAGAAA-CAC-GTTTCTGGTAAGA-GCTGCGTTTGGA-CATTT-CTCAGTGAA-TGGGAGTAT-ATGC-TGT
34  TAGATTG-AGGAGTTAGAAA-CAC-GTTTCTGGCAATG-GCTGACTTT-GGACATTCC-TGAGTGAATTGG-AGTAT-ATAC-TGT
35  TAGATTC-AGGAGATAGAAA-CAC-ATTCCTGGCAAGT-GCTGCGTTT-GGACATTCC-TGAGTTTATTGG-AGTAT-ATAC-TGT
36  TAGATTC-AGGAGTTAGAAA-CAC-GTTTCTGGCAAGA-GCTGCATTT-GGACATTCC-GGAGTGAATGGG-AGTAT-ATGC-TGT
37  TAGATTC-AGAAGTTAGAAT-CAC-GTTTCTAACCAGA-GCTGCGTTT-GGACATTCC-TGAGTGAATGGG-AGTAT-ATGC-TGT
38  TAGATTC-AGGAGTTAGAAA-CAC-GATTCTGCTAAGA-GTTGCCTTTGCA-CATTT-CTCAGTTAA-TGGGAGCCT-ATTG-TGT
39  TAGATTC-AGGAGTTAGAAAACAC-GTTTCTGCTAAAA-GCTGCGTTTGGA-TATTT-CTCAGTGAA-TGGGAGCCT-ATGC-TGT
40  TAGTTTC--AGCATTCGAAA-CAA-GTTTATTCTAGGA-GCTGCGTTTTAA-TATTC-CAGAGTGAA-TGGGAGTACACTGCGT--
41  TAGATTC-AGGAGTTAGAAA-CAC-GTTTCTGGTAAGA-GCTGCGTTTGGA-CATTT-CTCAGTGAA-TGGGAGCCT-ATGC-TGT
```

Figure S1, page 12 of 12

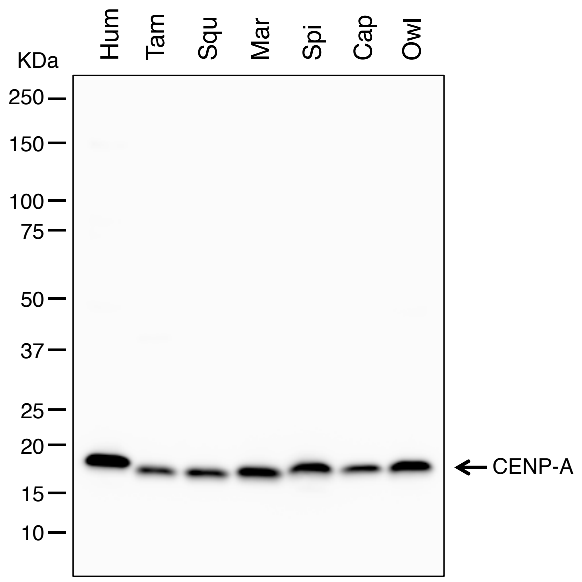
Hum
Tam
Squ
Mar
Spi
Cap
Owl
KDa
250
150
100
75
50
37
25
20
15
10
CENP-A

Figure S2
